# Probing Intracellular Element Concentration Changes during Neutrophil Extracellular Trap Formation Using Synchrotron Radiation Based X-Ray Fluorescence

**DOI:** 10.1371/journal.pone.0165604

**Published:** 2016-11-03

**Authors:** Björn De Samber, Maria J. Niemiec, Brecht Laforce, Jan Garrevoet, Eva Vergucht, Riet De Rycke, Peter Cloetens, Constantin F. Urban, Laszlo Vincze

**Affiliations:** 1 Department of Analytical Chemistry, Ghent University, Ghent, Belgium; 2 Department of Clinical Microbiology / MIMS, Umeå University, Umeå, Sweden; 3 Microbial Immunology Research Group, Hans Knöll Institute / Leibniz-Institute for Natural Product Research and Infection Biology, Jena, Germany; 4 DESY, Hamburg, Germany; 5 Inflammation Research Centre, VIB and Department of Biomedical Molecular Biology, Ghent University, Ghent, Belgium; 6 Department of Plant Systems Biology, VIB and Department of Plant Biotechnology and Bioinformatics, Ghent University, Ghent, Belgium; 7 European Synchrotron Radiation Facility, Grenoble, France; Hospital for Sick Children, CANADA

## Abstract

High pressure frozen (HPF), cryo-substituted microtome sections of 2 μm thickness containing human neutrophils (white blood cells) were analyzed using synchrotron radiation based X-ray fluorescence (SR nano-XRF) at a spatial resolution of 50 *nm*. Besides neutrophils from a control culture, we also analyzed neutrophils stimulated for 1–2 *h* with phorbol myristate acetate (PMA), a substance inducing the formation of so-called Neutrophil Extracellular Traps (or NETs), a defense system again pathogens possibly involving proteins with metal chelating properties. In order to gain insight in metal transport during this process, precise local evaluation of elemental content was performed reaching limits of detection (LODs) of 1 *ppb*. Mean weight fractions within entire neutrophils, their nuclei and cytoplasms were determined for the three main elements P, S and Cl, but also for the 12 following trace elements: K, Ca, Mn, Fe, Co, Ni, Cu, Zn, Se, Br, Sr and Pb. Statistical analysis, including linear regression provided objective analysis and a measure for concentration changes. The nearly linear Ca and Cl concentration changes in neutrophils could be explained by already known phenomena such as the induction of Ca channels and the uptake of Cl under activation of NET forming neutrophils. Linear concentration changes were also found for P, S, K, Mn, Fe, Co and Se. The observed linear concentration increase for Mn could be related to scavenging of this metal from the pathogen by means of the neutrophil protein calprotectin, whereas the concentration increase of Se may be related to its antioxidant function protecting neutrophils from the reactive oxygen species they produce against pathogens. We emphasize synchrotron radiation based nanoscopic X-ray fluorescence as an enabling analytical technique to study changing (trace) element concentrations throughout cellular processes, provided accurate sample preparation and data-analysis.

## Introduction

Neutrophils, the most frequent type of white blood cells, are circulating cells of the innate immune system serving as first line of defense against microbial pathogens [1]. They are terminally differentiated, non-proliferate cells armed with a large antimicrobial arsenal [2]. Neutrophils track and hunt microbial invaders by means of chemotactic migration towards the intruders: specialized receptors enable them to recognize microbes and to launch their antimicrobial program. The process is characterized by the phagocyte flowing around the pathogen and engulfing it into a phagocytic vesicle, fusing with lysosomes releasing their lethal content: reactive oxygen species (ROS), nitric oxide, antimicrobial proteins/peptides and (metal) binding proteins. In contrast to conventional phagocytosis an additional, extracellular defense mechanism was recently discovered. Upon stimulation, neutrophils are able to release so-called Neutrophil Extracellular Traps (or NETs) into the extracellular milieu, ensnaring and killing microbes [3–7]. NETosis refers to a form of pathogen induced cell death as opposed to different cellular death programmes such as apoptosis or necrosis [8, 9]. The formation of NET-structures is believed to be in close connection with the removal of essential trace elements from the pathogens, which is referred to as ‘nutritional immunity’, possibly involving proteins with metal chelating properties. In this way, NETs form a defense mechanism against microbes using chelating proteins, removing crucial trace elements from the pathogen. Metals such as Mn, Fe, Co, Ni, Cu and Zn are therefore high-probability candidates for interaction with NETs.

Gaining insight at the complex spatial distribution of these trace level elements during NET-formations can nowadays be achieved via a few select nanoscopic analytical techniques, such as nano-secondary ion mass spectrometry (nano-SIMS) [10] and synchrotron radiation nanoscopic X-ray fluorescence (SR nano-XRF) [11, 12]. Fluorescent dyes are also generally used for metal imaging in cells, but generally offer only microscopic resolution and sometimes have low sensitivity. Moreover, some dyes interfere with biological processes, are only sensitive to the free unbound metal ion, or additionally to other elements than the one under consideration [13–16]. Other trace level analytical techniques such as inductively coupled plasma mass spectrometry (ICP-MS) are powerful in terms of sensitivity, but in case of liquid analysis isolation procedures of target elements are prone to contamination and by using laser ablation systems spatial resolution is restricted to the single micrometer level [17]. Synchrotron radiation induced X-ray fluorescence distinguishes itself due to (ultra) trace level sensitivity (down to 100 *ppb* in imaging mode) with superior sub-micrometer resolution (currently at the 10 *nm* scale), deep penetrating nature, low susceptibility for contaminations and non-destructive character. Recently, also novel X-ray imaging techniques have become available such as X-ray phase contrast tomography providing morphological information on different length scales from organ to sub-cellular level [18, 19] and X-ray based scanning coherent diffraction imaging (CDI), also known as ptychography, capable of morphological imaging with a resolution of about 10 *nm* [20, 21] and currently being used to investigate the function of nanoscopic objects and materials within single cells [22, 23].

**Fig 1:**
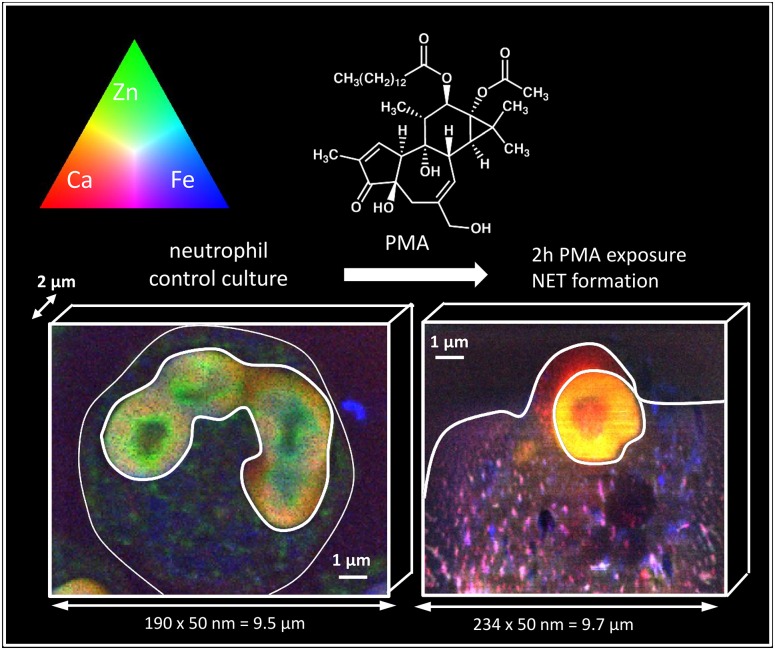
RGB composite images of the trace level element distribution of Ca, Zn and Fe (represented by the red, green and blue color channel respectively) of two single, high pressure frozen and cryosubstituted human neutrophils (white blood cells) before and after stimulation with phorbol myristate acetate (PMA), inducing the formation of so-called Neutrophil Extracellular Traps (or NETs). NETs are newly discovered structures which are believed to act as a defense mechanism against microbes via chelating proteins, removing crucial trace elements from the pathogen. As all intensities in the trichromatic maps are normalized to a single upper value, increases in color brightness also represent increases in concentration. A clear increase in cellular Ca concentration is revealed via appearance of a yellow nucleus and reddish cytoplasm. Intracellular Fe and Fe/Ca-rich structures are also emerging after 2 *h* stimulation with PMA, visible as blue and pinkish hot-spots. The absent green color in the PMA-stimulated neutrophil indicates the strong association of Zn to Ca and/or Fe. Results were obtained using synchrotron radiation based X-ray fluorescence at the ID22NI beamline (European Synchrotron Radiation Facility, Grenoble) operating at a spatial resolution of 50 *nm*.

In previous research, we investigated the trace elemental properties of human neutrophils—resting and activated—by nanochemical XRF imaging of forming NETs induced by phorbol myristate acetate, abbreviated to PMA in what follows. PMA is a phorbol esther that activates protein kinase C (PKC) at *nM* concentrations *in vitro* and *in vivo* [24]. The latter protein is a central regulator for many effector functions related to neutrophils. PMA stimulation leads to activation of NADPH oxidase and consequently ROS production, induces endocytic uptake, degranulation and triggers the processes which cumulate in the release of NETs [5]. We performed analyses upon freeze-dried neutrophils over 100 *μm* long distance, but also upon high-pressure frozen and cryosubstituted neutrophils generally confined within a 10 by 10 μm^2^ area. Two different third generation synchrotron sources were used: PETRA III in Hamburg and ESRF in Grenoble, respectively [25]. Fig 1 shows trichromatic maps (also referred to as RGB composite images) of the trace level element distribution of Ca, Zn and Fe (represented by the red, green and blue color channel, respectively) of two single, human neutrophils before and after stimulation with PMA (for Lewis structure see also Fig 1), until now the most potent agent to induce NET formation. Results were obtained using synchrotron radiation based X-ray fluorescence at the ID22NI beamline (European Synchrotron Radiation Facility, Grenoble) operating at a spatial resolution of 50 *nm*. Before the SR experiment, cell cultures were high pressure frozen, cryosubstituted in Spurr’s resin and cut with a microtome into 2 *μm* thin sections, which are schematically represented in Fig 1. As all intensities in the trichromatic maps are normalized to a single upper value, increases in color brightness also represent increases in concentration, revealing sub-micrometer quantitative element information within the neutrophils. We observe a clear increase in cellular Ca concentration via the emergence of a yellow nucleus and reddish cytoplasm. Intracellular Fe and Fe/Ca-rich structures visible as blue and pinkish ‘hot-spots’ after 2 *h* PMA stimulation appear as well. The absent green color in the PMA-stimulated neutrophil indicates the strong association of Zn to Ca and/or Fe.

As a continuation of this SR-XRF based study upon neutrophils, we initially study the spatial distribution, co-localization and quantitative changes of the trace elements Ca, Zn and Fe throughout PMA-stimulation by means of scaled RGB composite maps in Results and Discussion (R&D), ‘Nanoscopic imaging of Ca, Zn and Fe within single neutrophils throughout PMA-stimulation’. Then we indicate how quantitative data is obtained for a *single* neutrophil for all 15 detected elements, i.e. P, S, Cl, K, Ca, Mn, Fe, Co, Ni, Cu, Zn, Se, Br, Sr and Pb in R&D, ‘XRF quantitative results on single neutrophils’. Finally, mean weight fractions are calculated for neutrophil replicates measured for each PMA exposure time in R&D, ‘Mean element weight fractions of neutrophils in function of PMA exposure time’. These weight fractions are not only calculated for entire neutrophils, but also for the straightforwardly distinguishable sub-areas of nucleus and cytoplasm. Although adequate quantification of (trace-level) elements obtained via SR-XRF has already been shown via different approaches (e.g. calibration methods [26, 27], fundamental parameter method [28–30] and Monte Carlo simulation [31, 32]), SR-XRF based research papers investigating quantitative element weight fractions of a large number of elements within single cells and their compartments under influence of an external stimulus in a time course are rather rare. Also, in most cases only a few element concentrations are discussed, whereas in the case of XRF a wide element range can be addressed simultaneously. Provided the large amount of data and quantitative information obtained, statistical analysis was used for objective interpretation of the results.

## Results and Discussion

### Nanoscopic imaging of Ca, Zn and Fe within single neutrophils throughout PMA-stimulation

**Fig 2:**
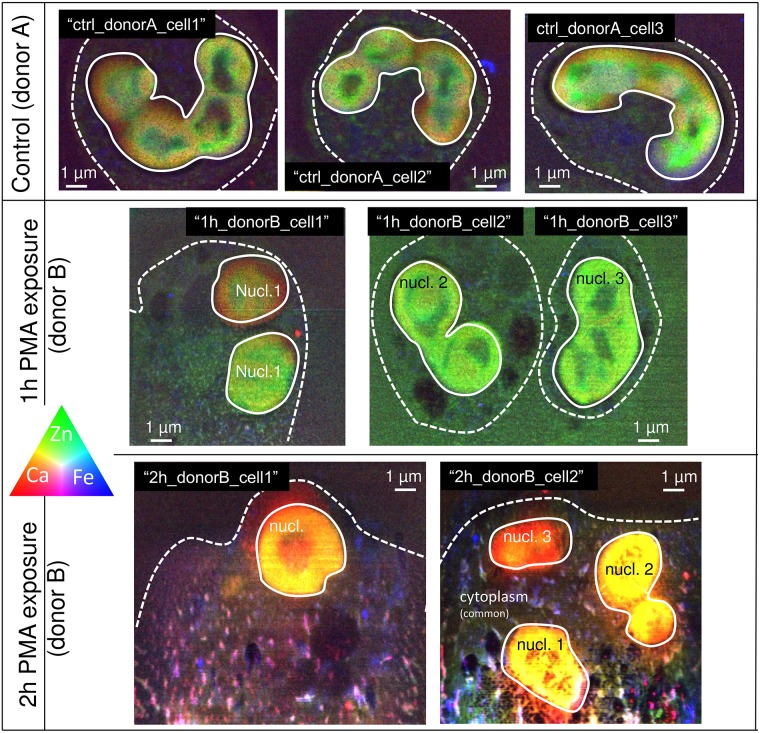
RGB composite element maps of all quantitatively assessed neutrophils throughout PMA-stimulation. The red, green and blue color channels represent the intensities of the Ca, Zn and Fe distribution, respectively. Upper row contains RGB composite element maps of neutrophils from control culture from donor A, middle and bottom row contain RGB composite element maps of neutrophils from donor B stimulated for 1–2 *h* with phorbol myristate acetate (PMA), respectively. All element intensities within the RGB composite element maps are normalized to diode current, dead time and dwell time per point (generally 300 *ms*). Maximum intensity of Ca, Zn and Fe (normalized) counts measured for the neutrophils from control culture were set as upper threshold values for the other RGB composite element maps. Within each RGB composite element map, the borders of nucleus and cytoplasm are indicated by a full and dashed white line, respectively. From each nucleus/cytoplasm area, an XRF sum spectrum was generated and used for quantification. Neutrophil nomenclature used in the quantitative analysis is provided within each RGB composite element map.

Fig 2 shows RGB composite maps of neutrophils from control culture, 1 *h* and 2 *h* PMA exposure obtained by SR-XRF. Color channels red, green and blue represent the elements Ca, Zn and Fe respectively. The use of these three elements in the RGB composite maps is motivated by different reasons: Zn exhibits high signal-to-noise ratios in its elemental maps, providing detailed morphology for most of the neutrophils. Calcium shows the largest intensity increase of all elements throughout stimulation with PMA (see R&D, ‘Mean element weight fractions of neutrophils in function of PMA exposure time’), whereas Fe showed a kind of changing speckle pattern. All elemental XRF intensities within the RGB maps are normalized to diode current (proportional to the incident beam intensity), detector dead time and dwell time (generally 300 *ms*). The maximum intensity of Ca, Zn and Fe for the neutrophils from control culture was set as an upper threshold, which was preserved for rendering the 1 *h* and 2 *h* PMA-stimulated neutrophil RGB composite maps. This allowed optimal observation of concentration changes of Ca, Zn and Fe throughout stimulation. Due to the limited amount of measuring time allocated at the beamline instrument, only a limited number of cells was scanned (approximately 20), which resulted in a maximum of 3 replicate neutrophil measurements per PMA exposure condition. For the same reason, neutrophils from only 2 different donors were measured. Neutrophils from control culture were only measured for donor A, whereas 1 *h* and 2 *h* PMA-stimulated neutrophils originate from donor B. The reason for this is that only satisfying elemental distributions for control neutrophils were found for donor B due to time restraint and lower quality/amount of cells present on a specific wafer. Therefore, the mentioning of donor A and B in Fig 2 is only indicative, i.e. for showing the donor origin of the neutrophils and not to stress the preferred experimental design.

The top row of Fig 2 contains 3 RGB composite element maps from control culture neutrophils, which are referred to as “ctrl_donorA_cell1”, “ctrl_donorA_cell2” and “ctrl_donor_A cell3”. The middle row shows RGB maps of neutrophils stimulated for 1 *h* with PMA: a single neutrophil cell “1h_donorB cell1” and two neighboring neutrophil cells “1h_donorB_cell2” and “1h_donorB_cell3”. The bottom row shows RGB maps of two neutrophil cells stimulated for 2 *h* with PMA: “2h_donorB_cell1” and “2h_donorB_cell2”. Within each RGB composite element map, cell border and nucleus are outlined with a dashed and full white line, respectively. These straightforwardly discernable areas were selected for quantifying their mean element content in an attempt to gain insight in metal fluxes in neutrophils throughout PMA-stimulation (discussed in R&D, ‘XRF quantitative results on neutrophil element content throughout PMA stimulation’). In this context, the three nuclei-like structures confined within the shared cytoplasm of “2h_donorB_cell2” are referred to as “2h_donorB_nucleus2.1”, “2h_donorB_nucleus2.2” and “2h_donorB_nucleus2.3”. As the indication of neutrophil nucleus and cytoplasm borders somehow masks the boundaries of these structures, RGB maps of the neutrophils without cell border and nucleus indication are provided in S1 Fig.

From the RGB composite element map, we clearly observe the lobulated structure of the control culture neutrophil nuclei, showing non-coinciding enrichments of Ca and Zn and the presence of perinuclear Fe, i.e. Fe located around the nucleus. In the cytoplasm of neutrophils from control culture, non-coinciding Zn/Fe-rich sub-regions are present, varying in size but well below the micrometer level. After 1 *h* PMA stimulation (Fig 2, middle row), we observe areas poor in elements detectable with XRF, which are most probably vacuoles. We also see a starting decondensation of the nucleus and disappearance of perinuclear Fe. After 2 *h* PMA stimulation (Fig 2, third row), we find an increased Ca concentration in the entire cell. In the nucleus, this concentration increase is spatially similar to the Zn distribution and therefore leading to its yellow color. The same is valid for the cytoplasm, leading to a uniform pinkish color with superimposed bright-pink Ca/Fe and blue Fe enriched hot-spots, respectively. We also observe a distinct increase of Fe-rich granules in the cytoplasm after 2 *h* PMA stimulation. Zinc is mostly present in a non-associated manner to Ca and Fe, both in nucleus and cytoplasm of neutrophils from control and 1 h PMA-stimulated culture, which leads to the bright green color of these RGB composite maps. After 2 *h* stimulation with PMA the dominant presence of Zn in green color disappears, not indicating its decrease in concentration, but rather the strong association of Zn with Ca and Fe, characterized by yellow and purple color, respectively.

We conclude that using scaled RGB composite maps is an ideal manner for studying co-localized element distributions of 3 elements throughout PMA-stimulation in a quantitative manner. In this way, we observed an increase of Ca in the neutrophil nucleus and cytoplasm in a co-localized manner with the distributions of Zn. Additionally, Ca/Fe and Fe-containing hot-spots appeared in the cytoplasm after 2 *h* PMA stimulation. Neutrophils stimulated for 3 *h* with PMA were also measured but were mainly composed of noisy element maps and therefore not included in Fig 2. We assume that neutrophils exposed for 3 *h* with PMA have diluted their cell content after cell burst in a larger volume; this causes the majority of concentrations to go (ultra) trace level and therefore below the level of detection. This phenomenon was also accompanied by increased sample radiation damage; for more information on this topic, we refer to our previous work [25]. Concerning the use of neutrophils originating from two different donors, we mention that our main aim of the study was to investigate changes from one PMA-stimulation times to another and not between neutrophils from different donors. Differences in neutrophil element distributions as a result of donor origin appeared to be negligible compared to the influence of PMA. Exploring metal fluxes in neutrophils throughout PMA stimulation could therefore only be achieved from the limited amount of donor and replicate cells and therefore not from a representative collection representing the average world population.

**Fig 3:**
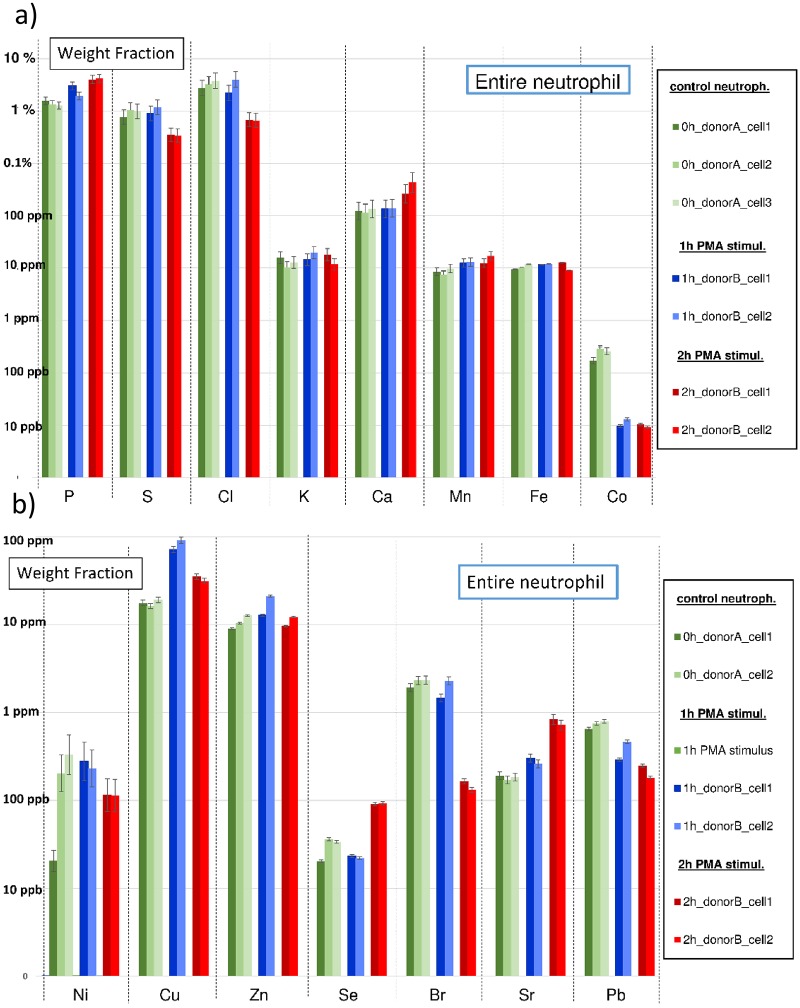
a-b: Bar graphs with element weight fractions within entire neutrophils throughout PMA stimulation for the elements P, S, K, Cl, K, Ca, Mn, Fe, Co (upper graph, Fig 3a) and for Ni, Cu, Zn, Se, Br, Sr and Pb (lower graph, Fig 3b). Neutrophils from control culture are indicated in green, 1 *h* PMA-stimulated neutrophils in blue and 2 *h* PMA-stimulated neutrophils in red. Weight fractions are expressed in *%*, *ppm* or *ppb* in a (different) logarithmic scale. Weight fraction values are normalized to the Compton intensity of neutrophil “0h_donorA_ cell1”. Error bars are based upon Poisson counting statistics and the certified uncertainty values of NIST SRM1577C “Bovine liver”.

Besides element maps of the elements Ca, Fe and Zn, also element maps were obtained for all other detectable elements. For the element maps of P, S, Ca, Mn, Fe, Cu and Zn, we refer to our previous work [25]; note that composite element maps “ctrl_donorA_cell1”, “1h_donorB_cell2-3” and “2h_donorB_cell2” in Fig 2 share a common data source with Fig 3a–3c therein. The obtained element maps were acquired with a dwell time per pixel of 300 *ms*, resulting limits of detection (LODs) of 0.96 *ppm* for Fe, 0.33 *ppm* for Zn and 0.11 *ppm* for Sr. More information concerning limits of detection achievable in scanning mode for other elements is provided in M&M (Materials and Methods), ‘Determination of limit of detection’ and S4 Fig. Trace elements present at concentrations close to their LODs, in our case Mn, Ni, Cu, Se, Sr and Pb therefore show poor contrast or contain only noise in their element distribution maps making quantitative comparison unreliable or impossible. In what follows, we shed light upon quantitative information of (ultra) trace elements within entire neutrophils, their nuclei and cytoplasms based on clustering of single point XRF spectra. Because the method can be generally applied, the analysis is not only performed for trace elements, but for all elements detected.

### XRF quantitative results on neutrophil element content throughout PMA -stimulation

#### XRF quantitative results on single neutrophils

XRF quantification was first performed upon the individual neutrophil cells; for more information on the general quantification procedure, i.e. from spectral fitting, normalization towards quantitative values we refer to M&M, ‘Single and batch fitting of XRF spectra’ to ‘Quantification of neutrophil XRF cluster spectra’. A flowchart describing the individual steps of the quantification procedure is provided in S1 Flowchart. As we believe that additional Compton normalization effectively eliminates irregularities between different samples, all quantifications were performed using the Compton normalized XRF intensities, more detail on this matter is provided in M&M, ‘Compton based normalization’. Besides XRF sum spectra of entire neutrophils, XRF cluster spectra of sub-areas nucleus and cytoplasm were generated and quantified as well. More information on the generation of XRF cluster spectra of entire neutrophils, nuclei and cytoplasms is provided in M&M, ‘Creation of nucleus and cytoplasm cluster sum spectra’.

S1 Table provides weight fractions of two (arbitrarily) chosen single neutrophils: one neutrophil from control culture “0h_donorA_cell2” and another one from a 2 *h* PMA-stimulated culture “2h_donorB_cell1”, which are also indicated in Fig 2. Weight fractions of the neutrophil sub-areas nucleus and cytoplasm are provided as well. For estimating the relative and absolute error of the concentrations, Poisson statistics and the certified concentration values of the reference material NIST SRM1577C were taken into account; more information is provided in S1 Text.

#### Mean element weight fractions of neutrophils in function of PMA exposure time

**Fig 4:**
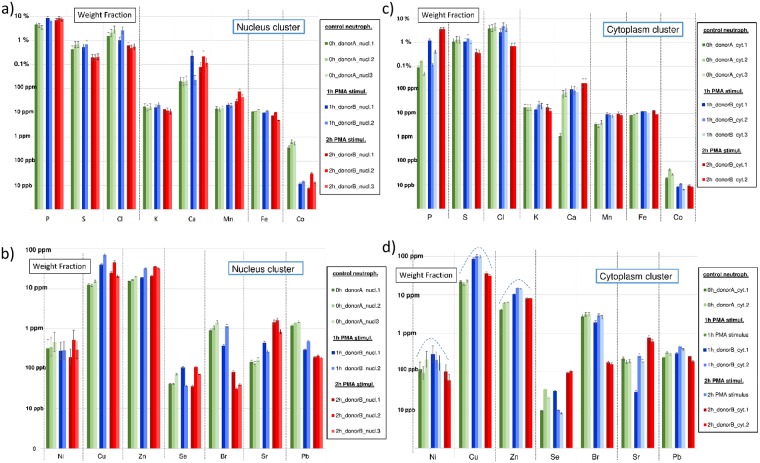
a-d: Bar graphs with element weight fractions within neutrophil nuclei (left column, Fig 4a-b) and cytoplasms (right column, Fig 4c-d) throughout PMA stimulation for P, S, K, Cl, K, Ca, Mn, Fe, Co (upper row) and for Ni, Cu, Zn, Se, Br, Sr and Pb (lower row). Neutrophils from control culture are indicated in green, 1 *h* PMA-stimulated neutrophils in blue and 2 *h* PMA-stimulated neutrophils in red. Weight fractions are expressed in *%*, *ppm* or *ppb* in a (different) logarithmic scale. Weight fraction values are normalized to the Compton intensity of neutrophil “0h_donorA_ cell1”. Error bars are based upon Poisson counting statistics and the certified uncertainty values of NIST SRM1577C “Bovine liver”.

The approach followed for obtaining weight fraction values within single neutrophils, their nuclei and cytoplasms is discussed in previous section. However, for more reliable interpretation of the results, also mean weight fraction values of a number of ‘replicate’ neutrophils having identical PMA-stimulation time were calculated and analyzed. The results of this analysis are discussed here. Bar plots showing the element weight fractions within the entire neutrophils throughout PMA stimulation are provided in Fig 3a for the elements P, S, K, Cl, K, Ca, Mn, Fe, Co and in Fig 3b for the elements Ni, Cu, Zn, Se, Br, Sr and Pb. Fig 4a–4d show weight fraction bar plots for the neutrophil nuclei (Fig 4a and 4b) and cytoplasms (Fig 4c and 4d) throughout PMA-stimulation. All weight fractions are expressed in *w%*, *ppm or ppb* on a (different) logarithmic scale. Weight fractions obtained from control culture are indicated in green, 1 *h* PMA stimulation weight fractions in blue and 2 *h* PMA-stimulation weight fractions in red bars. Weight fractions were normalized to the Compton scatter of entire neutrophil cluster “0h_donorA_cell1”, containing 22780 pixels and a having a dead time corrected Compton intensity of 3.91×10^7^ *cts*. All concentration values were calculated using a Spurr’s resin density of 1.13 *g/cm*^*3*^.

Corresponding quantitative numbers of Figs 3 and 4 are provided in Table 1. As multiple neutrophils were scanned per PMA-exposure duration, ±1×RSD value is given for each mean weight fraction value. Note that here, defined uncertainty ranges are larger compared to the single cell quantitative results presented earlier as RSD value is based upon weight fraction variation between the different cells. Whenever ±1×RSD range is larger than 25% of the concentration value, calculated weight fractions and the associated uncertainty ranges are in italics. To make results accessible to a wider readership, quantitative results of Table 1 are converted to other familiar concentrations used in other fields of science such as molarity (e.g. in *nM*, *μM* or *nM*) and areal concentrations (e.g. in *mg/cm*^*2*^, *μg/cm*^*2*^ or *ng/cm*^*2*^). Absolute element masses within the neutrophil (compartments) is provided as well (e.g. in *pg*, *fg*, *ag*); all the discussed converted quantities are provided within S3 Table.

**Table 1:** Mean weight fraction of P, S, Cl, K, Ca, Mn, Fe, Co, Ni, Cu, Zn, Se, Br, Sr and Pb within entire neutrophils, their nuclei and cytoplasms from control culture and 1–2 h PMA-exposed culture.

Entire neutrophil				Nucleus			Cytoplasm		
	control (n = 3 cells)	1 h PMA (n = 3 cells)	2 h PMA (n = 2 cells)	control (n = 3 nuclei)	1 h PMA (n = 3 nuclei)	2 h PMA (n = 4 nuclei)	control (n = 3 cytoplasms)	1 h PMA(n = 3 cytoplasms)	2 h PMA (n = 2 cytoplasms)
**P**	1.40 ± 0.14%	*2*.*33 ± 0*.*88%*	3.69 ± 0.18%	9.29 ± 1.33%	*12*.*9 ± 4*.*11%*	15.9 ± 1.9%	*0*.*196 ± 0*.*133%*	*0*.*772 ± 1*.*02%*	6.43 ± 0.73%
**S**	0.938 ± 0.155%	0.784 ± 0.072%	0.312 ± 0.029%	1.36 ± 0.32%	1.13 ± 0.07%	0.415 ± 0.01%	2.29 ± 0.39%	*1*.*44 ± 0*.*65%*	*2*.*08 ± 2*.*11%*
**Cl**	3.26 ± 0.52%	2.44 ± 0.33%	0.603 ± 0.004%	*4*.*87 ± 1*.*47%*	*3*.*45 ±1*.*13%*	*1*.*38 ± 0*.*49%*	7.64 ± 0.91%	*4*.*20 ± 1*.*73%*	*0*.*572 ± 0*.*785%*
**K**	13.0 ± 2.6 ppm	14.2 ± 1.0 ppm	13.5 ± 3.7 ppm	40.1 ± 2.8 ppm	34.5 ± 1.8 ppm	26.1 ± 2.1 ppm	34.5 ± 2.6 ppm	*22*.*1 ± 9*.*50 ppm*	*547 ± 744 ppm*
**Ca**	125 ± 11 ppm	117 ± 18 ppm	*316 ± 117 ppm*	470 ± 30 ppm	*0*.*183 ± 0*.*254%*	*0*.*264 ± 0*.*150%*	*91*.*4 ± 77*.*4 ppm*	*112 ± 64 ppm*	*163 ± 220 ppm*
**Mn**	8.66 ± 1.17 ppm	11.1 ± 2.0 ppm	13.4 ± 3.3 ppm	34.2 ± 1.9 ppm	37.5 ± 6.8 ppm	*90*.*9 ± 55*.*9 ppm*	6.88 ± 0.80 ppm	*10*.*1 ± 5*.*5 ppm*	*169 ±220 ppm*
**Fe**	*10*.*6 ± 3*.*4 ppm*	9.48 ± 1.35 ppm	9.86 ± 2.25 ppm	28.0 ± 3.5 ppm	20.4 ± 1.5 ppm	*14*.*1 ± 6*.*7 ppm*	17.5 ± 2.1 ppm	*13*.*8 ± 7*.*4 ppm*	*7*.*86 ± 10*.*6 ppm*
**Co**	*241 ± 62 ppb*	9.3 ± 0.6 ppb	8.8 ± 0.6 ppb	*1*.*18 ± 0*.*32 ppm*	25.4 ± 2.8 ppb	*30 ± 27 ppb*	*59 ± 30 ppb*	*10*.*2 ± 5*.*6 ppb*	*29 ± 22 ppb*
**Ni**	*187 ± 158 ppb*	*200 ± 54 ppb*	103 ± 2 ppb	878 ± 176 ppb	509 ± 79 ppb	*592 ± 413 ppb*	*254 ± 104 ppb*	*270 ± 190 ppb*	*10*.*2 ± 14*.*3 ppm*
**Cu**	17.8 ± 1.5 ppm	64.0 ± 2.9 ppm	30.0 ± 1.8 ppm	30.6 ± 4.1 ppm	103 ± 15 ppm	*57*.*3 ± 30*.*4 ppm*	39.2 ± 0.7 ppm	*107 ± 46 ppm*	*32*.*7 ± 26*.*2 ppm*
**Zn**	10.8 ± 1.9 ppm	15.2 ± 3.5 ppm	9.90 ± 1.90 ppm	40.0 ± 5.8 ppm	48.0 ± 6.5 ppm	*54*.*9 ± 21*.*6 ppm*	10.4 ± 2.8 ppm	*14*.*5 ± 5*.*8 ppm*	*6*.*68 ± 9*.*09 ppm*
**Se**	30 ± 9 ppb	18.1 ± 3.5 ppb	83.0 ± 3.5 ppb	< LOD	*111 ± 104 ppb*	*144 ± 73 ppb*	*42 ± 27 ppb*	*23 ± 25 ppb*	*212 ± 57 ppb*
**Br**	2.21 ± 0.25 ppm	*145 ± 109 ppb*	134 ± 18 ppb	2.62 ± 0.61 ppm	*1*.*55 ± 0*.*65 ppm*	*103 ± 44 ppb*	5.59 ± 0.75 ppm	*2*.*70 ± 1*.*11 ppm*	*1*.*70 ± 2*.*05 ppm*
**Sr**	183 ± 9 ppb	*212 ± 63 ppb*	703 ± 57 ppb	350 ± 34 ppb	*587 ± 309 ppb*	*2*.*75 ± 0*.*75 ppm*	362 ± 28 ppb	*158 ± 142 ppb*	*613 ± 549 ppb*
**Pb**	739 ± 78 ppb	30.7 ± 1.7 ppb	194 ± 38 ppb	3.13 ± 0.35 ppm	714 ± 60 ppb	388 ± 57 ppb	517 ± 111 ppb	*422 ± 184 ppb*	*152 ± 215 ppb*

Weight fractions are expressed in *%*, *ppm or ppb*. The number of clusters used for calculating each mean concentration and relative standard deviation (RSD) per exposure condition is given at the top of each column. Uncertainty is expressed as ±1×RSD of the number of cells measured for each condition. Whenever ±1×RSD range is larger than 25% of actual weight fraction value, calculated weight fractions and the associated uncertainty ranges are in italics.

Table 2 provides statistical analysis performed upon the obtained quantitative data using SPSS 23^™^ statistics software package (IBM). First, normality of the distribution ‘concentration *vs* time’ was first verified by a P-P plot of the regression standardized residual. We found the weight fraction values of each element throughout PMA stimulation to be normally divided, therefore both linear regression and analysis of variance (ANOVA) could be applied. As for some elements normal distribution was better than others, elements were also classified according to their degree of normality. The slope ß_0_ of the obtained linear regression curve provides an important quantity to assess metal concentration changes in neutrophils and their compartments throughout PMA-stimulation time, which can be expressed in e.g. *w%*, *ppm or ppb per hour*. In cases of poor linearity (*r*^*2*^ < 0.5), ß_0_ values and the corresponding 95% confidence interval are omitted; whenever *r*^*2*^ ≥ *0*.*5*, values are indicated in bold. For the cases most different from normal distribution, a non-parametric Kruskal-Wallace (KW) test was performed additionally. When the significance value for both tests (ANOVA and KW) is close to 0, the null hypothesis ‘values not significantly different’ can be rejected. In this case the significance value is indicated in bold, in the other case in italics.

**Table 2:** Statistical data-analysis upon mean weight fractions of entire neutrophils (a), their nuclei (b) and cytoplasms (c) throughout PMA stimulation.

		P	S	Cl	K	Ca	Mn	Fe	Co	Ni	Cu	Zn	Se	Br	Sr	Pb
**ENTIRE CELL**	P-P plot linear	+	+++	++	+	++	++	+++	++	+	+	+++	++	+++	++	+++
	lin regr. *r*^*2*^	**0.785**	**0.808**	**0.868**	*0*.*015*	**0.521**	**0.566**	*0*.*069*	**0.703**	*0*.*101*	*0*.*121*	0.000	**0.476**	**0.945**	**0.722**	**0.866**
	lin regr. β_0_ [*ppb/*h]	1.13E+07	-3.01E+06	-1.29E+07		8.76E+04	2.40E+03	-4.40E+02	-1.24E+02				2.30E+01	-1.02E+03	2.42E+02	-2.85E+02
	trend	+	-	-	const.	+	+	const.	-	-	const	const.	+	-	+	-
	95% conf. Int. Lower	5400992	-4474798	-17959782		-84866	302	-2055	-206				-1	-1260	92	-397
	95% conf. Int. Upper	17224381	-1542530	-7879792		130617	4495	1176	-43				48	-771	392	-173
	ANOVA F-value	21.925	25.214	39.345	0.091	6.522	7.834	0.444	14.196	0.676	0.826	0.003	5.394	103.224	15.575	38.679
	ANOVA sign.	**0.003**	**0.002**	**0.001**	*0*.*773*	**0.043**	**0.031**	*0*.*53*	**0.009**	*0*.*442*	*0*.*398*	*0*.*961*	**0.059**	**0.000**	**0.008**	**0.001**
	KW test χ^2^	4,694			,556				5,833	1,806	6,250		5,361		4,048	
	KW test sign.	**,096**			,*757*				**,054**	,*405*	**,044**		**,069**		,*132*	
**NUCLEUS**	P-P plot linear	+++	+	++	+++	+	++	+++	+	+++	++	+++	+++	++	+	++
	lin regr. *r*^*2*^	**0.61**	**0.824**	**0.733**	**0.9**	*0*.*287*	*0*.*357*	**0.665**	**0.698**	*0*.*15*	*0*.*071*	*0*.*197*	*0*.*027*	**0.869**	**0.737**	**0.827**
	lin regr. β_0_ [*ppb/*h]	3.30E+07	-4.82E+06	-1.76E+07	-7.08E+03		2.94E+04	-6.91E+03	-5.50E+02	-1.33E+02	1.08E+04	7.42E+03	1.30E+01	-1.27E+03	1.24E+03	-1.33E+03
	trend	+	-	-	-	+	+	-	-	-	+	+	const.	-	+	-
	95% conf. Int. Lower	1.15E+07	6.63E+06	-2.63E+07	-9.01E+03		-2.80E+03	-1.09E+04	-8.46E+02	-3.91E+02	-2.09E+04	-4.79E+03	-5.10E+01	-1.67E+03	6.36E+02	-1.82E+03
	95% conf. Int. Upper	5.45E+07	-3.00E+06	-8.94E+06	-5.16E+03		6.17E+04	-2.91E+03	-2.55E+02	4.43E+02	4.25E+04	1.96E+04	7.60E+01	-8.66E+02	1.85E+03	-8.32E+02
	ANOVA F-value	12.513	37.536	21.945	72.121		4.435	15.911	18.492	1.411	0.614	1.966	0.219	53.289	22.391	38.286
	ANOVA sign.	**0.0080**	**0.000**	**0.0020**	**0.000**		**0.068**	**0.004**	**0.003**	*0*.*269*	*0*.*456*	*0*.*198*	*0*.*652*	**0.000**	**0.001**	**0.000**
	KW test χ^2^					3,682					6,564			8,018	8,018	8,018
	KW test sign.					,*159*					**,038**			**,018**	**,018**	**,018**
**CYTOPLASM**	P-P plot linear	+	++	++	+	++	+	++	+	+	+	+++	+++	+++	++	++
	lin regr. *r*^*2*^	**0.741**	*0*.*019*	**0.88**	*0*.*283*	*0*.*078*	*0*.*317*	*0*.*317*	*0*.*255*	*0*.*292*	*0*.*002*	*0*.*201*	**0.527**	**0.678**	*0*.*082*	**0.497**
	lin regr. β_0_ [*ppb/*h]	2.92E+07		-3.53E+07	2.36E+05	3.47E+04	7.52E+04	-4.72E+03	-1.80E+01	4.59E+03	2.22E+03	-1.41E+03	7.70E+01	-2.02E+03	1.00E+02	-1.76E+02
	trend	+	const.	-	+	+	+	-	-	+	const	-	+	-	+	-
	95% conf. Int. Lower	11947643		-48291558	-139326	-84309	-35180	-11633	-48	-2556	-51676	-8294	4	-3407	-234	-352
	95% conf. Int. Upper	46495877		-22249123	610738	153707	185633	2201	12	11736	56112	5470	150	-628	434	0.89
	ANOVA F-value	17.134	0.116	43.929	2.365	0.509	2.78	2.783	2.081	2.47	0.01	0.252	6.69	12.626	0.536	5.927
	ANOVA sign.	**0.006**	*0*.*745*	**0.001**	*0*.*175*	*0*.*502*	*0*.*147*	*0*.*146*	*0*.*199*	*0*.*167*	*0*.*923*	*0*.*634*	**0.041**	**0.012**	*0*.*492*	0.051
	KW test χ^2^	4,250		6,250	2,889		3,222		4,421	,111					3,806	
	KW test sign.	,*119*		**,044**	,*236*		,*200*		,*110*	,*946*					,*149*	

P-P plot shows the degree of normality: ‘+++’ indicates perfect alignment of data points with *y = x*, ‘++’ larger distance from data points to *y = x* and ‘+’ large distance from data points to *y = x*, asymmetry and presence of ‘staircases’. Linear regression coefficients *r*^*2*^ and ß_0_ (in *ppm per hour*) are displayed, as well as lower and upper limit of the 95% confidence interval. Variance analysis (ANOVA) was performed showing F-value and significance. In case of less ideal normal distribution, a non-parametric Kruskal-Wallace (KW) test was performed, providing χ^2^ and (asymptotic) significance value. *r*^*2*^ values ≥ 0.5 and significance values for ANOVA and KW test close to 0 (= values significantly different) are indicated in bold, otherwise in italics

For clarity, we discuss the different elements with increasing atomic number, i.e. we first address the lighter elements P, S, Cl, K and Ca, followed by the transition metals Mn, Fe, Co, Ni, Cu and Zn, non-metals Se and Br and finally the heaviest detected elements Rb, Sr and Pb. Lighter elements such as P, S and Cl are present in weight percentages, show normal distribution throughout PMA-stimulation and, after linear regression, show good *r*^*2*^ values in the 0.6–0.8 range for the entire neutrophil cell, nuclei and cytoplasms. Phosphorus shows a concentration increase of approx. +1.13 *w% per hour*, rising from 1.40 *w%* to 3.69 *w%*. As this increase was also observed to a greater extent in the nucleus (up to 15.9 *w%*) and to a lesser extent in the cytoplasm, these findings suggest an extracellular uptake of P throughout PMA-stimulation. Sulphur, on the other hand, shows a linear decrease of -0.30 *w% per hour* in entire neutrophils; a larger concentration decrease of -0.48 *w%* is found for the nucleus and a rather constant one for the cytoplasm, which suggests a release of S from the decondensing nucleus and additional extracellular release of S. Note that for this element, we generally find a higher concentration in the cytoplasm than in the nucleus: 2.29 *w%* vs 0.94 *w%* in neutrophils from control culture. Yarom et al. also measured mean element concentrations in human lymphocytes using electron microscopic X-ray microanalysis and obtained quantitative numbers of 4.10 ± 0.09 *w%* and 0.66 ± 0.03 *w%* for P and S in human neutrophils respectively [33], which are of the same order as the weight fraction values of 1.40 ± 0.14 *w%* and 0.938 ± 0.155 *w%* obtained in our study. Note however that we detected a considerable amount of S (and Cu) in the Spurr’s resin, which could be a potential source of influence; for more information on this matter we refer to M&M, ‘Composition of Spurr’s embedding resin’ and S2 Table. For Cl, we observe (like for P) a cellular concentration decrease of -1.3 *w% per hour*; the effect is however stronger for the cytoplasm than for the nucleus (-3.5 *w% vs* -1.8 *w% per hour*). The decrease of Cl present in nucleus and cytoplasm throughout PMA-stimulation may be related to the fact that Cl uptake is required for/occurs under activation of NET forming neutrophils [34]. Chlorine present inside the cell could be made available for NET formation in areas outside the cell, and therefore cause a decrease in intracellular Cl concentration. A low *r*^*2*^ value of 0.015 is indicative for a rather constant K (potassium) weight fraction in entire neutrophils of approx. 13.5 *ppm*. We found however a clear linear (*r*^*2*^ = 0.9) decrease of K concentration in the nucleus of -7.1 *ppm per hour*, which is likely to be associated with the K concentration increase in the cytoplasm of +236 *ppm per hour*. For Ca, generally present in a tenfold higher concentration than K, a fairly linear (*r*^*2*^ = 0.52) concentration increase in (entire) neutrophils cells throughout PMA-stimulation was found of +88 *ppm/hour*; for nucleus and cytoplasm rather poor *r*^*2*^ values were obtained but concentrations are also increasing. Mean Ca concentrations in entire neutrophils are rising from 125 to 316 *ppm*; concentrations in the nucleus rise even from 470 *ppm* to weight percentage levels of 0.26 *w%*. The overall increasing Ca concentration throughout PMA-stimulation can be explained by its uptake via the activation of Ca channels during NET formation; hereby is Ca present in relatively high concentration in the medium [35]. Similarly, ROS-independent forms of NETosis require calcium fluxes [36]. Besides Ca, also a fairly linear (*r*^*2*^ = 0.57) concentration increase in the entire neutrophils was observed for Mn. Since this element is present in concentrations more similar to K in our case (a tenfold lower than Ca), a (much) lower concentration increase of +2.4 *ppm per hour* is obtained. For Mn in nucleus and cytoplasm, an increasing concentration was observed for both these cell compartments, suggesting an uptake of this element from the extracellular environment under PMA-stimulation. Manganese is considered as a nutrient that is required for many pathogens to establish an infective lifestyle and sequestered by neutrophil calprotectin [37]. In absolute terms, we found the Mn concentration in the entire neutrophils gradually increasing from 8.7 to 13.4 *ppm* after 2 *h* PMA stimulation and in the nuclei even from 34.2 to 90.9 *ppm* (for the cytoplasm uncertainty intervals were too large to draw sound conclusions). Although showing poor linearity in concentration and having a slowly declining concentration in entire neutrophils throughout PMA stimulation, Fe shows a linear (*r*^*2*^ = 0.67) weight fraction decrease in the nucleus of -6.9 *ppm per hour*. The declining concentration could be related to the formation of Fe rich hot-spots in the cytoplasm forming after 2 h PMA stimulation. Also the decondensation of the nucleus throughout PMA stimulation results in a release of its content to the environment, causing a diluting effect. For Co, a good linear (*r*^*2*^ = 0.7) decrease in concentration throughout stimulation is observed in entire neutrophils (-124 *ppb per hour*) and nuclei (-550 *ppb per hour*). In absolute numbers, this translates to a neutrophil mean concentration decreasing from 241 to 9 *ppb* and nucleus mean concentration from 1.18 *ppm* to 30 *ppb;* cytoplasm concentration values are staying rather constant around 30 *ppb*. The reason for the high Co concentration in neutrophils from control culture, coupled with large concentration decrease upon stimulation remains unclear.

For the elements Ni, Cu and Zn poor linearity throughout PMA stimulation is observed. Remarkably, we discerned for our specific study a peculiar recurrent pattern in the concentration for these elements (and for Pb) throughout PMA-stimulation, generally showing higher concentration values towards 1 *h* PMA-stimulation. The pattern, indicated by a dashed line in Fig 4c, is best recognizable and shows large resemblance for the areas of cytoplasm and background. The fact that the pattern is present for different elements present in concentration ranges of different orders of magnitude could indicate a sample-specific dilution effect. When referring to the sample preparation procedure in M&M, ‘Sample preparation’, culture medium is washed away with cold (5*°C*) ultrapure water in order to remove the heavy salt matrix from the neutrophils as it generates unwanted XRF signal. Such manually performed procedure, includes in all likelihood variations. As the recurrent pattern is less often observed for the nucleus than for cytoplasm weight fractions, it could be an indicator that the nucleus is less affected by the washing procedure than the cytoplasm. We believe that during the washing procedure the nucleus can be considered as a rather sturdy ‘chemical container’ compared to the more easily penetrable cytoplasm. Also, the specific element for which the fingerprint occurs can provide information which elements are more sensitive to dilution and/or leaching during the washing step, i.e. Cu, Zn, Ni (and Pb) in our case. When looking at Table 1, we see that Ni is generally present below the *ppm* level, i.e. in the 100–200 *ppb* range for the entire cell, whereas Cu and Zn in the 10–100 *ppm* range for entire neutrophil, nuclei and cytoplasm. Concentration levels for Cu are similar in nucleus and cytoplasm, whereas Zn has a fourfold higher concentration in the nucleus of up to 55 *ppm*. The Zn concentration most likely bears close resemblance to the real world case whereas the Cu concentration is likely increased by its high concentration in the Spurr’s resin (see M&M, ‘Composition of Spurr’s embedding resin and S2 Table). Although Ni, Cu, Zn are thus likely influenced during the sample preparation procedure, these elements are known to play a prominent role in many biological processes and therefore we cannot exclude these elements from playing a role during NET-formation. In contrast to Ni, Cu and Zn, Selenium shows a linear (*r*^*2*^ = 0.5, both cell compartments) concentration increase for the entire neutrophils and cytoplasm of +23 and +53 *ppb per hour*, most pronounced after 2 *h* PMA stimulation. Se trace level concentrations vary in the 20–200 *ppb* range, close to the 140 *ppb* LOD in scanning mode (see M&M, ‘Determination of limits of detection’). As Se is known for its antioxidant activity, its increased presence may be a protective measure of neutrophils against their own so-called reactive oxygen species (or ROS) produced against pathogens [38]. Bromine shows an excellent linear decrease (*r*^*2*^ = 0.95) in concentration over the entire neutrophil of -1.02 *ppm per hour*; which is also observed in nucleus and cytoplasm, suggesting a release of this element throughout PMA-stimulation. Note that this element is present in a (slightly) lower concentration range than previous elements Mn, Fe and Zn, i.e. 2–5 *ppm* in neutrophils from control culture down to 100 *ppb* for 2 *h* PMA exposure. The declining concentration of Br is most likely linked to Cl due to the identical valence and similar chemical properties of both elements (see above). Since the deployed 17 *keV* excitation energy is close to the Sr K-edge at 16.1 *keV*, the LOD for Sr is as low as 1 *ppb* for a live time of 3000 *s*, which is the typical integrated measuring time for a neutrophil sub-area such as a nucleus or cytoplasm (see M&M, ‘Determination of limits of detection’). We observe a linear (*r*^*2*^ = 0.7) increase of +240 *ppb per hour* within the entire neutrophils and an increase of +1.24 *ppm per hour* of Sr in their nuclei, corresponding to concentration increases from 180 to 700 *ppb* and from 350 *ppb* to 2.8 *ppm*, respectively. As was the case for Br, the concentration increase of Sr is most likely linked to the chemically similar element Ca. For Pb, we observe a linear concentration decrease throughout PMA stimulation of -285 *ppb per hour*, resulting in a weight fraction decrease from 740 *ppb* to 194 *ppb* in entire neutrophils. Such decrease could, as was the case for Fe, be related to the decondensation of the nucleus, evoking a dilution effect. Due to the similar trend pattern of Cu, Ni and Zn that is also observed for Pb and its presence in beamline (shielding) components we disregarded the obtained results for Pb in this study.

Summarizing, by creating clusters of the entire neutrophils, nucleus and cytoplasm sub-compartments, XRF sum spectra with high peak-to-background ratio could be obtained. After rigorous normalization of these cluster sum spectra, Fundamental Parameter (FP) quantification was performed, allowing quantification of all 15 detected elements. Due to the longer measuring time associated with the neutrophil cluster areas, element sensitivities are increasing a hundredfold, providing element concentrations at the *ppm* and *ppb* level. For the most sensitive elements in our experimental set-up, limits of detection (LODs) are reaching the single *ppb* level. We found weight fractions of most of the detected elements to be normally divided, which allowed linear regression analysis providing concrete numbers of concentration changes throughout PMA stimulation. For a majority of elements, linear concentration changes in function of PMA exposure (time) were found. Subdividing the neutrophil in sub-compartments such as nuclei and cytoplasm provided additional insight whether metal concentration changes are still occurring linear and/or if metal exchange is occurring intra- or extracellular. Practically, for P, S and Cl, concentration changes in the *w% per hour* range were found throughout PMA stimulation; this with highest concentration increase for P (+1.13 *w% per hour*) and highest concentration decrease for Cl (-1.30 *w% per hour*). The decreased Cl concentration has been related to the extracellular requirement of Cl during NET formation by Nizet et al. [34]. For Ca, we found an increasing concentration in entire neutrophils of *+88 ppm/hour*, most likely related to its uptake via the activation of Ca channels during NET formation [35]. In this respect, we found a rather constant K concentration (13 *ppm*) in entire neutrophils throughout NET-formation, although a clear linear decrease throughout stimulation was observed in the nucleus of *-7*.*1 ppm per hour*. For poor contrast or noise maps of trace level elements Mn, Ni, Cu Se, Sr and Pb our cluster method provided additional ‘hidden’ information on concentration changes of these elements throughout stimulation. Manganese for example, shows an overall increase in concentration at a much lower rate of 2.4 *ppm per hour*, suggesting an extracellular uptake from the environment. The concentration increase could be related to the sequestration of this metal by the protein calprotectin to prevent it from being harvested by pathogens [37]. Fe showed a linear (*r*^*2*^ = 0.67) weight fraction decrease in the nucleus of -6.9 *ppm per hour*, probably related to decondensation of the nucleus throughout stimulation and release of its content in the extracellular surroundings. For elements such as Ni, Cu and Zn, poor linearity throughout PMA stimulation was observed. Owing to its multi-element detection capability, sample-specific element ‘fingerprints’ were found for these elements, in our opinion caused by sample preparation differences. Interestingly, our analysis not only shows which *elements* but also which *cell compartments* are more susceptible to this, in our case the cytoplasm. In order to circumvent this shortcoming, cryogenic sample preparation and analysis is suggested (see also Summary & Conclusions). Cobalt showed a surprisingly high concentration in the nuclei of 240 *ppb*, rapidly decreasing to a tenfold lower values after 1–2 *h* PMA stimulation. Increased Co intensities were measured for 3 neutrophils from control culture. Although external reasons such as sample contamination are not excluded, a phenomenon of biological nature could also be a reason behind. Selenium shows a linear concentration increase (*r*^*2*^ = 0.5) for the entire neutrophils of +*23 ppb per hour* and cytoplasm, especially visible after 2 *h* stimulation. This concentration increase might be related to the known antioxidant activity of this element and its possible protective role against reactive oxygen species (or ROS) that neutrophils release against pathogens [38]. For Br and Sr, we observed a linear decrease and increase in concentration throughout PMA-stimulation of *-1*.*0 ppm* and *+240 ppb per hour* respectively, also observed for nucleus and cytoplasm in similar concentrations ranges; which can most likely be assigned to the identical valence and similar chemical properties of the element pairs Br/Cl and Sr/Ca.

## Materials and Methods

### Sample preparation

As a complete description of the sample preparation procedure has already been published [25] (see also Introduction), we provide here only a concise summary of the sample preparation procedure of the neutrophils, focusing on steps which may influence the element concentrations of neutrophils during the sample preparation procedure. To isolate neutrophils from peripheral blood we have acquired venous blood from 2 healthy voluntary donors with informed written consent in accordance to an ethical permission 09-210M from the regional ethical board in Umeå, Sweden. The 2 donor blood samples are further referred to in the manuscript as A. & B. (these abbreviations are also used in Fig 2). The blood samples exclusively served as a source for primary neutrophils. No donor data were used or saved anywhere, nor were any personal data relevant for the study. The blood was sampled by trained personnel, not by the authors themselves. Neutrophils were isolated by means of centrifugation of a 1:1 layered fresh blood/histopaque 1119 and suspended in conventional medium solution. Cells were then seeded on a sapphire disk, used for high pressure freezing. PMA stimulus was added and cells were incubated to adhere onto the support. The cell medium was removed and cells were washed twice with ultrapure water to remove the cell medium, containing salt and interfering trace metals. The washing procedure was performed briefly (5 *s* duration) and with cold (5*°C*) water in order to prevent (osmotic) cell burst. As a required step prior to HPF, 50 *m*L of 20*% w/v* BSA in PBS (Sigma) was added onto each sapphire disk as a cryoprotectant. Note that cells were in contact with the BSA-containing PBS only very briefly to avoid element exchange between cell and medium. The sapphire disk was then quickly high pressure frozen (HPF) and cryosubstitution was performed with acetone and 0.1% glutaraldehyde. Later on, samples were warmed up and embedded in Spurr’s resin. Sections of approx. 2 *μm* thickness were cut and finally deposited on silicon nitride membranes of 3.0 x 3.0 *mm* membrane size, 500 *nm* membrane thickness, 7.5 x 7.5 *mm* frame size and 200 *mm* frame thickness (Silson Ltd, Northampton, UK). For quantification of the neutrophil element content, NIST SRM 1577C ‘bovine liver’ was selected. A total mass of 17.4 *mg* was pressed in a self-supporting pellet, resulting in an areal density of 13.1 *mg/cm*^*2*^. This type of biological reference material is particularly well-known for its excellent homogeneity on the (sub-) micrometer scale [39].

### Composition of Spurr’s embedding resin

Element weight fraction values of the Spurr’s resin only are provided in S2 Table. In particular we found, both for the elements S and Cu, a higher weight fraction in the Spurr’s resin than in the neutrophils, which for both elements can most likely be traced back to the used cryoprotectant bovine serum albumin (BSA). BSA is a protein with high molecular weight applied upon the neutrophils during high pressure freezing (HPF). As this protein contains 640 Sulphur atoms out of 68.574 atoms in total, it may contribute to the determined S weight fraction of 6.7 ± 1.3 *w%* in the Spurr’s resin containing the neutrophils from control culture (S weight fraction in the Spurr’s resin containing 1 *h* and 2 *h* PMA-exposed neutrophils amounts 7.0 ± 0.2 *w%* and 6.0 ± 1.1 *w%*, respectively). The high Cu concentration of 140 *ppm* in the Spurr’s resin containing the neutrophils from control culture on the other hand may be caused by contamination of BSA with trace metals during its production process as the absence of trace metals is not guaranteed by the manufacturer (Sigma Aldrich). Another plausible reason is the presence of chemically bound trace metals since albumin is known to have 5 Zn and 1 Cu binding position; some of the amino acids in albumin are also known to become phosphorylated. These findings strongly encourage the application of other cryoprotectants than BSA for future XRF studies on HPF treated cells; cryoprotectants less likely to contain (trace level) metals comprise 1-hexadecene, sucrose or gelatin.

### Experimental set-up

The scanning nano-XRF experiments were performed at the former ID22NI (nano-imaging) beamline at the European Synchrotron Radiation Facility (ESRF), which was installed at a high-β straight section equipped with two different undulators covering an energy range of 6–70 *keV* [40]. The instrument was dedicated to hard X-ray nanoanalysis, allowing nano-XRF and absorption/phase contrast nanotomography and is currently replaced by the NINA project (nano-imaging and nano-analysis), available at ID-16A and ID-16B beamlines, respectively. X-ray focusing was obtained by a crossed elliptical Rh coated graded-multilayer mirror-pair in the Kirkpatrick–Baez (KB) geometry. X-rays are collected and focused in both vertical and horizontal axis at a glancing angle of 3.5 *mrad*. The first mirror, coated with a graded multilayer plays both the role of vertical focusing device and monochromator, resulting in a very high flux (exceeding 10^11^ *photons/s*) and a medium mono-chromaticity (*ΔE/E* 10^−2^). For collecting X-ray fluorescence emanating from the irradiated area, a silicon drift detector (SDD) was used (Vortex EX, Hitachi High-Technologies Science America Inc.). A collimator with narrow opening covers the detector nozzle, which is positioned as close as possible to the sample (both in the *mm* range). In this way, fluorescent radiation is collected only from the area irradiated with the X-ray beam and scatter radiation from the environment is rejected. The microtome sections are scanned preferably perpendicular to the beam as the focal plane of the optical microscope is used to bring the sample in the focus of the X-ray beam. Ideally the detector is put in the ring plane, under 90° with respect to the incoming beam, minimizing Compton scatter radiation. By geometrical constraints, the detector is put under a 15° angle with respect to the sample surface in order to collect X-ray fluorescent radiation. A digital pulse processor (microDXP, XIA LLC, Hayward CA, USA) was used for analyzing the signal generated by the SDD detector. A diode was installed behind the sample for determining the incoming flux density. In order to determine beam size (FWHM), knife-edge scans upon a Au test pattern were performed and provided a beam size of 64 *nm* vertically and 54 *nm* horizontally at an excitation energy of 17 *keV*. The latter allowed an efficient excitation and detection of K-fluorescent lines for the elements P up to Sr (and additionally the Pb L-lines).

### Data-analysis

#### Creation of nucleus and cytoplasm cluster maps and generation of the associated XRF sum spectrum

For each scan containing one (or more) cells, a black-and-white cluster map was created whereby the black pixels belong to the entire neutrophil cell, abbreviated to ‘*Ce*’ in what follows. Selection of the pixels belonging to the entire neutrophil was achieved by means of manual selection using a brush tool in Adobe Photo Shop CC (64 bit) using the clear drop in the *Zn*—*K*_*α*_ intensity map at the border between the neutrophil and the Spurr’s resin. Similarly, a cluster map of the nucleus ‘*Nucl*’ was created based on a clear *Zn*—*K*_*α*_ intensity increase at the transition between cytoplasm and nucleus. The cytoplasm area ‘*Cyt*’ is then obtained mathematically by subtracting the nucleus map ‘*Nucl*’ from the entire cell ‘*Cell*’ pixel map (see Eq 1.1). In a similar way, the matrix cluster map ‘Backgr’ was obtained by subtracting the entire cell cluster map ‘Cell’ from the entire scanned area ‘*Total’* (see Eq 1.2). All generated ‘cluster maps’ were then saved in a single datafile (*.ims), also containing the element (and scatter) maps. For each generated cluster map, i.e. entire cell, nucleus, cytoplasm and background, a sum spectrum was created by summing the individual point spectra belonging to the ‘cluster map’ in question.

Cyt=Cell−Nucl (1.1)

Backgr=Total−Cell (1.2)

S2 Fig shows cluster sum spectra of the entire cell (red), cytoplasm (green) and nucleus (blue) cluster maps obtained from the chosen (reference) single neutrophil from control culture “0h_donorA_cell1”, left-hand side graph. All spectra were normalized to the Compton signal of the nucleus cluster sum spectrum in order to make comparison possible. Black-and-white figures on the right-hand side are cluster maps of the cell, cytoplasm, nucleus and background/matrix, used for creating their corresponding cluster sum spectra (selected pixels are depicted in white). Pixel selection for cluster areas was based upon the neutrophil Zn-K_α_ distribution, shown in the lower right corner. Although XRF cluster sum spectra only provide information on the mean concentration of the cluster area they represent, they correspond to measuring times of approx. 50 *min*, resulting in sensitivities as much as 100 times lower than those in scanning mode (e.g. 300 *ms* in dwell time mode *vs* 3000 *s* for a cluster spectrum. For elements detected with high sensitivity like Sr, this results in LODs reaching the single *ppb* level. For more information on LODs of all detected elements under scanning mode (300 *ms* dwell time), single point (300 *s*) or cluster mode (3000 *s*), we refer to M&M ‘Determination of limits of detection’.

#### Single and batch fitting of XRF spectra

In order to obtain the ‘raw’ (i.e. not corrected for flux and dead time) net and background intensities of the SRM and (sample) cluster XRF sum spectra, individual XRF point spectra of each map (e.g. 2550 single point spectra with 500 *ms* dwell time in case of the SRM) were summed using the IDL programming-data visualization software (Harris corp., Florida, US). The software package AXIL (Analysis of X-ray Spectra using the Iterative Least Squares method) [41] was used for accurate fitting of the of a reference cluster sum spectrum of an entire neutrophil from control culture ‘0h_donorA_cell1’. Hereby the following aspects are taken into account: 1) selection of the spectral region of interest (ROI), 2) energy calibration (in *eV*/channel) 3) adding fluorescent lines of the detected elements, including escape and sum peaks 4) selection of background model, e.g. orthogonal polynomials 5) definition of Compton and Rayleigh scattering peak. In our case, following elements were included: K-lines of P, S, Cl, K, Ca, Mn, Fe, Co, Ni, Cu, Zn, Se, Br, Rb and Sr and L-lines of Pb (separately fitted). Detector sum peaks and escape peaks for all elements up to Zn were included as well. As the input model properly fitted all cluster sum spectra from NIST SRM 1577C and neutrophil samples, it could be used for both, simplifying the quantification procedure (sometimes it occurs that additional elements are present within the samples/SRM or that a different background model is required for optimal fit). Using the MICROXRF2 software package, a batch fit was performed for all individual point spectra of the recorded XRF sample maps using the single, above described fitting model. Finally, all total K_α_ net element and background intensities were saved.

#### Normalization of SRM spectral intensities

Element yields were calculated from the measurement on NIST SRM1577C. High flux density at the ID22NI beamline (~10^12^ *photons/s*) resulted in a too high dead time when measuring the standard. A 1500 *μm* Al absorber, providing a transmission of 12.5*%* at 17 *keV*, resulted in a flux reduction approx. by a factor of 8. A 10 x 10 *μm*^*2*^ area, randomly selected on the SRM, was scanned with a step size of 200 *nm* and 500 *ms* dwell time, resulting in a total measuring (real) time of 1275 *s* (21 *min* 15 *s*). The mean absolute diode current value when measuring the SRM was determined as 1.583 x 10^7^. Due to the insignificant sample absorption at the incident beam energy, the measured current can be regarded as being proportional with the incident beam intensity. For the SDD detector, both incoming and outgoing count rates were registered during the measurement, from which a mean deadtime value of 11.27*%* was deduced. As element yields were calculated from the SRM using a 1500 *μm* absorber in the beam, they necessarily need to be converted to the sample measurement conditions (i.e. no absorbers in the beam), requiring an additional correction. A ‘cluster map’ containing only pixels belonging to an entire neutrophil from control culture “0_donorA_cell1” was generated and from its corresponding sum spectrum, a reference mean diode current value (*I*_*0*_) of 1.511 x 10^8^ was calculated. For more information on the generation of a so-called ‘cluster map’, we refer to M&M, ‘Creation of nucleus and cytoplasm cluster XRF spectra’. Note that the reference mean diode current value is approx. 9.52 times higher than the mean diode current measured for the SRM, somewhat more than the factor 8 derived in the beginning of this paragraph.

The raw (element-specific) net *K*_*α*_ fluorescent line intensities of the SRM ‘$I_{K_{\alpha },\;raw}\left(SRM\right)$’ were then corrected for dead time ‘*DT(SRM)*’ and normalized to the mean diode current value of the chosen reference cluster sum spectrum ‘$\bar{k_{diode}\left(cluster_{ref}\right)}$’:

$$\;I_{K\alpha ,\;norm.}\left(SRM\right)=I_{K_{\alpha },\;raw}\;\left(SRM\right)*\;\frac{1}{1-DT\left(SRM\right)}*\frac{\bar{k_{diode}\left(cluster_{ref.}\right)}}{\bar{k_{diode}\left(SRM\right)}}$$ (2)

In our study the cluster sum spectrum was generated from an entire neutrophil from control culture ‘0h_donorA_cell1’. Thereafter, the normalized net *background* intensities of the SRM, required for calculation of the limits of detection (LODs) were calculated in the same manner, see M&M ‘Determination of limit of detection’.

#### Self-absorption correction of SRM by means of the Fundamental Parameter method

Quantification was based on a fundamental parameter approach which exploits the theoretical relation between the net fluorescent line intensities and the elemental concentrations [42]. This method requires that, before determining the element yields and limits of detection (LODs) from the SRM net fluorescent and background intensities, the (element-specific) absorption correction factor A_corr._ is calculated first:

$$A_{corr}=\frac{1-e^{-\chi \varphi d}}{\chi \varphi d}$$ (3)

in which φd is the so called “areal mass” of the sample (expressed in *mg/cm*^*2*^ in our case) and the absorption correction factor χ is calculated using:

$$\chi =e^{-\left(\frac{\mu_{0}}{\text{sin}\alpha }+\frac{\mu_{1}}{sin\beta }\right)}$$ (4)

in which μ_0_ represents the total cross-section of the SRM at the incoming beam energy of 17 *keV* and μ_1_ the total cross-section of the SRM at the energy of the fluorescent line of the element considered. Total element cross-sections for NIST SRM 1577C were calculated from the X-ray lib database [28, 43], called from IDL. The angles α and β represent the incident angle of the incoming beam upon the sample and the angle between sample and detector, in our case 90° and 15°, respectively. The absorption correction factor χ in function of the atomic number Z is shown in S3 Fig. The absorption correction factor is significant for low Z elements like P, S and Cl (0.686, 0.484 and 0.455, respectively), while for higher Z elements like Ni, Cu and Zn the correction factor is approaching 1 (i.e. 0.897, 0.914 and 0.929, respectively).

#### Determination of limits of detection (LODs) and absolute element yields

By using the A_corr_ factor calculated previously, the sensitivity (e.g. in *ppm*) of the experimental set-up can be estimated by calculating the relative LOD:

$$LOD_{relative}=\frac{1}{A_{corr}}\;.\;\frac{\sqrt{{\text{I}}_{\text{back},\text{ norm}.}}}{I_{K_{\alpha },\;norm.}}\;.\;conc_{\left(SRM\right)}$$ (5)

where $I_{K_{\alpha },\;norm.}$ and I_back,norm._ are the normalized fluorescent net *K*_*α*_ and background intensities of the SRM, respectively; *conc*_(*SRM*)_ is the certified concentration of the element in consideration of the SRM. The absolute element yield ‘*Yield*_*abs*._’ (e.g. in counts/(s * ag)), required for the quantification of (trace) elements in the neutrophil cluster sum spectra, can be calculated using the following formula:

$$Yield_{abs.}=\;\frac{1}{A_{corr.}}\frac{I_{K_{\alpha },\;norm.}}{\left(\varphi d\right).(conc._{SRM}).\left(\sigma_{hor.}.\sigma_{vert.}\right)}$$ (6)

in which *φd* is the areal mass of the standard (13.1 *mg/cm*^*2*^) and σ_hor._ and σ_vert._ are the horizontal and vertical FWHM (full width at half maximum) of the beamsize (64 *nm* horizontally by 54 *nm* vertically, respectively). S4 Fig shows in the upper graph the relative LODs obtained during the experiment at beamline ID22NI (Grenoble, ESRF) using no beam absorbers. Values are calculated for 1) a typical dwell (or scan) time of 300 *ms*, 2) a typical (live) 300 *s* long point measurement and 3) the measuring time for a typical cluster area (approx. 3000 *s*). Relative MDLs are calculated from *K*_*α*,*total*_ net line and background intensities, corrected for self-absorption effects (incident angle between incoming beam and sample surface is 90°, angle between sample surface and detector is 15°). The lower graph shows the absolute element yields (expressed in counts/(s*ag)) with and without absorption correction. For both graphs, vertical error bars are added; further information on the error estimation of the LOD and element yield is provided in S1 Text.

#### Quantification of neutrophil XRF cluster sum spectra

In order to obtain quantitative concentrations of a cluster map termed as ‘*cluster*_*x*_’ (e.g. entire cell, nucleus, cytoplasm or matrix/background), the ‘raw’ intensities of the cluster sum spectra need to be corrected for (mean) dead time and normalized to the mean diode current value of the reference cluster sum spectrum ‘*cluster*_*ref*_’, i.e. the XRF sum spectrum of an entire neutrophil cell from control culture “0h_donorA_cell1”:

$$\;I_{K\alpha ,\;conv.\;norm.}\left(cluster_{x}\right)=\frac{I_{K_{\alpha },\;raw}}{1-\bar{DT\left(cluster_{x}\right)}}\frac{\bar{k_{diode}\left(cluster_{ref.}\right)}}{\bar{k_{diode}\left(cluster_{x}\right)}}$$ (7)

Embedding the neutrophils in Spurr’s resin followed by cutting in 2 μm thin sections with a microtome provides a (near) constant sample thickness (and density), which greatly simplifies the quantification process. A setback of this simplification is the introduction of an additional quantity, i.e. sample thickness variable, with an associated uncertainty.

For determining the weight fraction of a certain element (e.g. in *%*, *ppm or ppb*), the total amount of mass *m*_*abs*._ (typically expressed in *pg*, *fg* or *ag*) present in *cluster*_*x*_ is first straightforwardly determined with the following formula:

$$m_{abs.}\left(cluster_{x}\right)=\frac{I_{norm}\left(cluster_{x}\right)}{Yield_{abs.}}$$ (8)

From the total mass of a certain (trace) element present within a cluster area, the weight fraction (typically expressed in *%*, *ppm* or *ppb*) can then be determined. First, the total mass of the matrix *m*_*matrix*_ is calculated by assuming a constant matrix density for the Spurr’s resin ‘*φ*_*Spurr*_’ of 1.13 *g/cm³* [44] and by estimating the total irradiated volume *V*_*Spurr*_. The latter is calculated by multiplying: 1) the number of pixels in the considered cluster *N*_*pix*._(*cluster*_*x*_), 2) the beam size *Area*_*beam*_ and 3) the estimated 2 μm thickness of the microtome sections *T*_*sect*._:

$$conc._{relative}=\frac{m_{abs.}\left(cluster_{x}\right)}{m_{matrix}}=\frac{m_{abs.}\left(cluster_{x}\right)}{\varphi_{Spurr}.V_{Spurr}}=\frac{m_{abs.}\left(cluster_{x}\right)}{\varphi_{Spurr}.N_{pix.}\left(cluster_{x}\right).Area_{beam}.{\text{ T}}_{\text{sect}.}}$$ (9)

All tabulated neutrophil weight fractions values in this paper are calculated using the density of Spurr’s resin in which the embedded cells were effectively measured. However, by assuming that the cell volume remains intact during the cryosubstitution process, replacement of the Spurr’s resin density (φ = 1.13 *g/cm³*) in Eq 9 by the lower density of water (φ = 1.0 *g/cm³*) delivers a 13% increased element weight fractions for the neutrophil *in vivo* condition. From the analyte mass within a cluster volume also the molar concentration can be calculated:

$$conc._{molar}\left(\frac{mole}{L}\right)=\frac{m_{absolute,\;cluster}}{M_{g}.V_{matrix}}=\frac{m_{absolute,\;cluster}}{M_{g}\;.(N_{pixels,\;cluster}.Area_{beam}.T_{sample})}$$ (10)

or the areal concentration (typically expressed in *μg/cm*^*2*^ or *ng/cm*^*2*^):

$$conc._{areal,\;cluster}\left(\frac{ng}{cm^{2}}\right)=\frac{I_{norm.,\;cluster}}{Yield_{areal}.\#\;pixels_{cluster}}$$ (11)

Note that in case of freeze-dried (or cryo-frozen) cells, the actual thickness (and therefore also the areal density) varies according to position. In that case the varying mass per unit area needs to be determined for accurate quantification. This quantity is however not straightforward to determine due to evaporation of low Z elements such as H, C and O during XRF analysis and lack of suitable reference material for mass calibration.

#### Compton based normalization

A Compton scatter peak is inherently present within each XRF spectrum and a measure for the so-called ‘irradiated sample mass’ of the analyzed area by the incident X-ray beam. When applying normalization procedure upon the raw Compton intensities of all measured neutrophil nuclei, a relative standard deviation (RSD) of 15% was found. This number corresponds in all probability to the variation of the illuminated sample mass by the incident beam. The illuminated sample mass varies most likely through differences in thickness between the different thin sections: during the cutting process, slice thickness is determined optically. The accuracy of such optical method has an estimated accuracy close to the 15% RSD value found. However, as differences in normalized Compton intensity are also present between different scanned neutrophils embedded within the same thin section, also other reasons must be responsible for the RSD value such as local differences in thickness and/or density within the same thin section. By assuming that the normalized (i.e. to dead time correction, mean diode current and cluster area) Compton intensities are largely caused by local variations in thickness and/or density, we can normalize the XRF net line intensities to the Compton intensity of a reference cluster sum spectrum, slightly modifying the formula previously used in M&M, ‘Quantification of neutrophil XRF cluster spectra’.

$$\;I_{K\alpha ,\;Compt.norm.}(cluster_{x}{)\;=\text{ I}}_{K\alpha ,\;\text{conv}.\text{ norm}.}\left(cluster_{x}\right).\frac{I_{Compton}\left(cluster_{ref}\right)}{I_{Compton}\left(cluster_{conv.norm}\right)}$$ (12)

More detailed information of the effect of Compton normalization upon the obtained weight fractions is provided in S2 Text and S5 Fig.

#### Remarks on Monte Carlo based quantification

We want to indicate that Monte Carlo simulation is another valuable tool for accurate quantification of biological material [31, 32]. However, in practice, a standard which is measured during the experiment is often used for calibrating the Monte Carlo simulation parameters (e.g. detector solid angle, absorbing layers) and therefore quantification by Monte Carlo simulation largely boils down to principles similar to the fundamental parameter approach. However, the Monte Carlo simulation provides the *entire* XRF spectrum of the sample/standard rather than discrete intensities, enabling the detection of additional scattering effects, such as so-called ‘enhancement effects’ (which are however of lesser importance in dilute systems like single cells) and also secondary scattering from the surrounding air, matrix or sample support.

## Summary and Outlook

Neutrophils play a crucial role in defending the human body against pathogens. By the formation of so-called Neutrophil Extracellular Traps (or NETs) they are able to withdraw and capture crucial metals such as Mn, Fe, Cu, Zn from the pathogen’s environment, presumably via metal chelating proteins, eventually starving the microbes to death. Such phenomena fit in the research field recently termed as ‘nutritional immunity’. We used synchrotron radiation X-ray fluorescence (SR-XRF) for trace level imaging of these (and other) metals within high pressure frozen, cryo-substituted and thin-sectioned human neutrophils from control culture on the one hand and neutrophils exposed to phorbol myristate acetate (PMA) on the other hand, a substance stimulating the formation of Neutrophil Extracellular Traps (or NETs).

SR based X-ray fluorescence analysis is a powerful method to visualize trace level metal distributions and provide quantitative upon regions of interest. Additionally, X-ray fluorescence analysis provides a kind of holistic approach in terms of element analysis in the sense that not a few elements are analyzed, but instead the whole range of elements excited under the experimental conditions. This multi-element character of SR based XRF is particularly useful when a wide range of elements are under investigation for possibly playing a prominent role in a biological phenomenon such as NET formation. RGB composite maps provided information concerning the co-localization of Ca, Zn and Fe throughout PMA-stimulation in a quantitative manner. We observed among others an increase of Ca in the neutrophil nucleus and cytoplasm in a co-localized manner with the Zn distributions. Additionally, Ca/Fe and Fe-containing hot-spots appeared in the cytoplasm after 2 h PMA stimulation. For several biologically relevant element maps, including Mn, Ni, Cu, Se and Sr, the element under consideration is present in concentrations close to or below the limit of detection associated with scanning mode. This results in poor contrast or even noise element maps, making it impossible to see clear differences throughout stimulation. In order to address this problem, we performed cluster analysis upon larger neutrophil areas. Precise local evaluation of P, S, Cl, K, Ca, Mn, Fe, Cu, Zn, Co, Ni, Se, Br, Sr and Pb weight fractions in resting and activated entire neutrophils was assessed by fundamental parameter based quantification. In an attempt to shed light upon intra- and extracellular mass fluxes, straightforwardly discernable sub-areas of nucleus and cytoplasm were quantified as well. Linear regression analysis indicated concentration changes throughout PMA-stimulation for P, S, Cl and the (ultra) trace elements K, Ca, Fe, Mn, Co and Se. The linear concentration increase for Mn could be related to scavenging of this metal from the pathogen by means of the neutrophil protein calprotectin [37], whereas the concentration increase of Se may be related to its antioxidant function protecting neutrophils from the reactive oxygen species they produce again pathogens [38].

Within this bio-analytically oriented work, we highlight SR based nano-XRF as an enabling analytical technique to investigate the possible role of a wide range of metals simultaneously for a certain biological phenomenon under scrutiny, in our case NET-formation in human neutrophils. Thanks to its multi-element character, it truly places other elements in the spotlight playing a potential role which would otherwise be left undisturbed, as in our case for the elements Cl, Ca, Mn, Fe, Co, Se, Br and Sr. Generally, other analytical techniques are probing only a limited number of elements which can *de facto* be analyzed simultaneously while maintaining sufficient sensitivity. In this way, SR based XRF also helped us in detecting sample-specific sample preparation artefacts for Ni, Cu and Zn; indicate similar behavior for chemically similar elements such as Ca-Sr and Cl-Br and finally probe trace level elements considered as more exotic in biology such as Co, Se and Sr. We communicate our findings to the broader research community, with the aim to help in the elucidation of the role of metals during NET-formation.

The use of inhibitors for possibly providing better internal control, suggested by Papayannopoulos et al. [45] was not considered in this study as we mainly focused on metal fluxes in neutrophils during PMA stimulation. Also, the use of inhibitors may also block the investigated metal fluxes, just as it is the case for NET formation/neutrophil activation. A mechanistic insight which could be controlled for using pharmaceutical inhibitors of different neutrophil activation pathways is not provided yet at this stage.

Considering future possibilities in this field of research, we foresee that sub-10 *nm* sized X-ray beams with unsurpassed flux above 10^12^ *photons/s* will provide exciting new possibilities in nanochemical imaging and quantitative multi-element analysis of single cell compartments, potentially reaching single atom detection level. High sample-throughput, in-vacuum cryogenic sample environments with idealized detector schemes will enable studies towards hundreds of cells per experiment and largely obliterate discussions on chemical fixation methods formerly used. Improvements in scanning algorithms and dedicated analysis software are expected to foster automatic cell recognition, followed by high-throughput scanning in a fully automated manner and quantitative (trace) level analysis of sub-cellular target areas such as nuclei, cytoplasms, lysosomes, vacuoles and mitochondria; combined with statistical analysis pointing out significant concentration differences.

## Supporting Information

S1 Fig RGB composite maps of all quantitatively assessed neutrophils. **RGB channels represent the intensities of the Ca, Zn and Fe distribution, respectively—neutrophil cell border and nucleus border are not shown here.** Upper row contains RGB composite element maps of control culture neutrophils from donor A, middle and bottom row contain RGB composite element maps of neutrophils from donor B stimulated for 1 *h* and 2 *h* with phorbol myristate acetate (PMA), respectively. All element intensities within the RGB composite element maps are normalized to diode current, dead time and measuring time. Maximum intensity of Ca, Zn and Fe (normalized) counts within the control culture neutrophils were set as upper threshold values for the other RGB composite element maps. Neutrophil nomenclature used in the quantitative analysis is provided within each RGB composite element map.
(TIFF)

S2 Fig XRF cluster sum spectra from entire cell (red), nucleus (blue) and cytoplasm (green). All XRF sum spectra (left hand side) are normalized to the Compton signal of the nucleus cluster sum spectrum of a single neutrophil from control culture ‘0h_donorA_cell1’. Black-and-white figures (right hand side) are corresponding cluster maps (selected pixels depicted in white). Selection of the different cluster areas was based upon the Zn-K_α_ element distribution (lower right corner).
(TIFF)

S3 Fig Calculated absorption correction factor, for a self-supporting NIST SRM 1577C ‘bovine liver’ pellet (13.1 *mg/cm*^*2*^) for all detected elements (parameters identical to the ID22NI geometrical configuration). Incident angle between incoming beam and sample surface is 90°, angle between sample surface and detector is 15°. Energy of the incoming beam was set to 17 *keV*. For the higher Z elements, a curve is added to ‘guide the eye’.
(TIFF)

S4 Fig relative limits of detection (LODs) (upper graph, Fig a), absolute element yields obtained at ID22NI beamline (lower graph, Fig b). MDLs are shown for a typical dwell time of 300 *ms* per point in scanning mode, a point measurement of approx. 300 *s* and a neutrophil sub-area with an approximate scanning time of 3000 *s*, without beam absorber. Element yields are shown with (blue color) and without (orange color) absorption correction factor. Relative LODs and element yields are both calculated from K_α,total_ net line and background intensities. Error bars are calculated from relative error of background and net intensity and relative concentration error of NIST SRM 1577C ‘bovine liver’; for more information on this matter, we refer to S1 Text.
(TIFF)

S5 Fig Effect of the normalization procedure upon the Compton scatter intensity. **Mass fractions of K, Mn and Fe within neutrophil nuclei and cytoplasms throughout PMA exposure obtained by ‘conventional’ and ‘Compton’ normalization.** *Right side of double vertical stripe*: effect of different normalization steps upon Compton intensity of XRF cluster sum spectra of neutrophil nuclei (above Y-axis) and cytoplasms (below Y-axis). First column: raw cluster sum spectra, 2^nd^ column: raw cluster sum spectra corrected for dead time (DT) and normalized to incoming beam intensity (I_0_), 3^rd^ column: additional normalization to cluster area (= number of pixels in reference cluster), 4^th^ column: cluster sum spectra normalized to the Compton intensity of the reference cluster. RSD values of nucleus and cytoplasm Compton intensities after each normalization step are shown in square boxes. *Left side of double vertical stripe*: comparison between K, Mn and Fe mass fractions of neutrophil nuclei (above Y-axis) and cytoplasms (below Y-axis) throughout PMA stimulation, calculated from ‘conventionally’ normalized (left bar) and ‘Compton’ normalized (right bar) element XRF intensities. Quantitative data is expressed in *ppm* and in logarithmic scale.
(TIF)

S1 Flowchart Quantification procedure followed for obtaining quantitative data on single neutrophils, their nuclei and cytoplasms throughout PMA stimulation. (TIF)

S1 Table weight fraction of P, S, Cl, K, Ca, Mn, Fe, Co, Ni, Cu, Zn, Se, Br, Sr and Pb within nucleus and cytoplasm of a single neutrophil from control culture “0h_donorA_cell2” and a *2 h* PMA-stimulated neutrophil “2h_donorB_cell1” respectively. (XLSX)

S2 Table Mean weight fraction of P, S, Cl, K, Ca, Mn, Fe, Co, Ni, Cu, Zn, Se, Br, Sr and Pb within the Spurr’s resin, expressed in %, *ppm* or *ppb* used for embedding neutrophils from control culture and 1–2 *h* PMA-exposed neutrophils. The number of clusters used for calculating each mean concentration and relative standard deviation (RSD) per exposure condition is given at the top of each column. Uncertainty is expressed as ± 1×RSD value of the number of cells measured for each condition.
(XLSX)

S3 Table Molar concentrations (a), areal concentrations (b) and absolute masses (c) of P, S, Cl, K, Ca, Mn, Fe, Co, Ni, Cu, Zn, Se, Br, Sr and Pb within entire neutrophils, their nuclei and cytoplasms from control culture and 1–2 *h* PMA-exposure. The number of clusters used for calculating each mean concentration and relative standard deviation (RSD) per exposure condition is given at the top of each column. Uncertainty is expressed as ±1×RSD of the number of cells measured for each condition. Areal concentrations of neutrophil cytoplasms were not provided as uncertainty ranges became too large.
(XLSX)

S1 Text Calculation of the relative error of 1) (absolute) element yield, 2) relative limits of detection (LODs), 3) absolute mass 4) concentration of the analyte + conversion of error bar values to log-scale. (DOCX)

S2 Text Comparison between weight fractions of K, Mn and Fe obtained by conventional and Compton normalization in PMA-stimulated neutrophils. (DOCX)
