# Supplementary figures and images for: Probing Intracellular Element Concentration Changes during Neutrophil Extracellular Trap Formation Using Synchrotron Radiation Based X-Ray Fluorescence
Source: PLoS One. 2016 Nov 3;11(11):e0165604. doi: 10.1371/journal.pone.0165604 (PMC5094720; doi:10.1371/journal.pone.0165604)

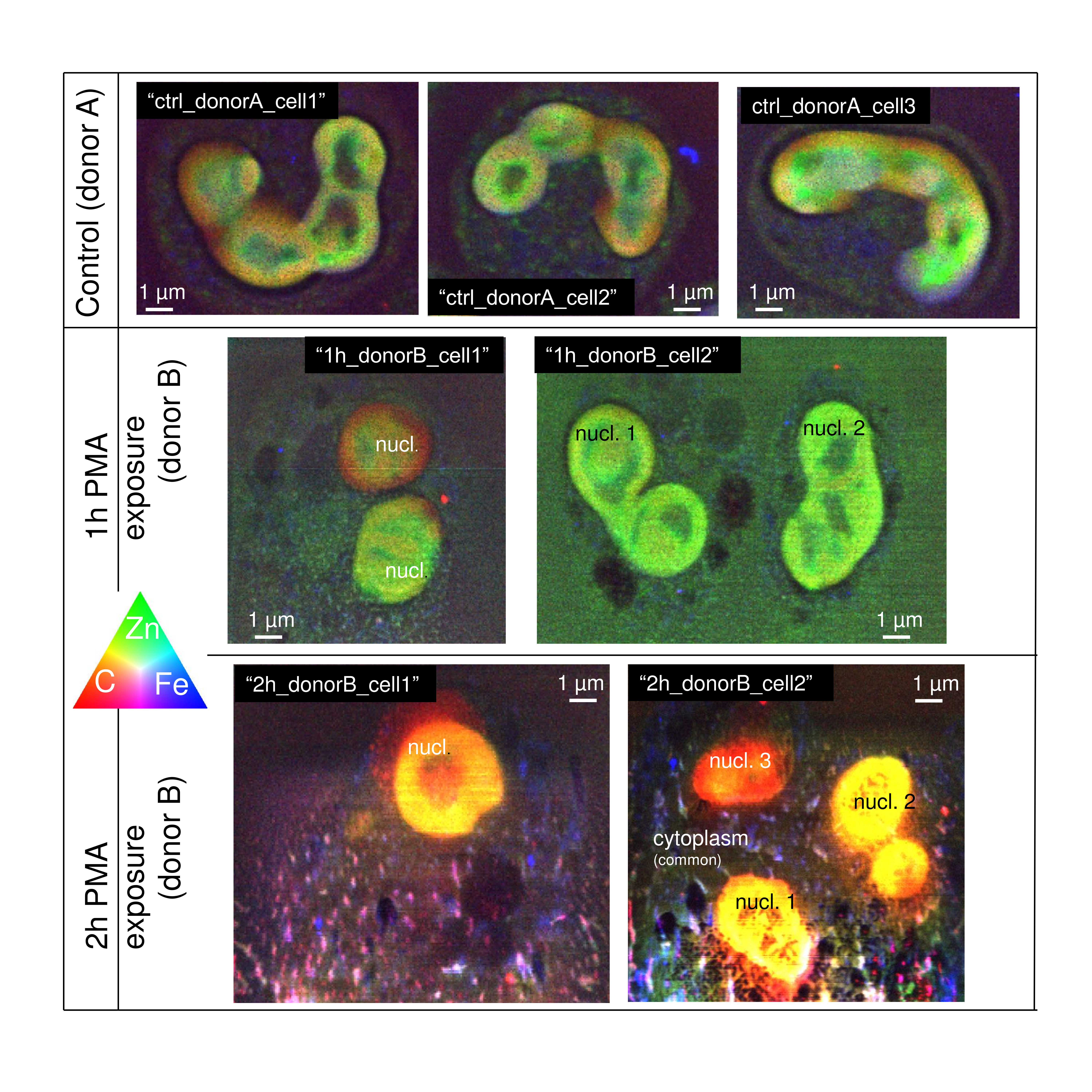

Supplement: S1 Fig — RGB channels represent the intensities of the Ca, Zn and Fe distribution, respectively—neutrophil cell border and nucleus border are not shown here. Upper row contains RGB composite element maps of control culture neutrophils from donor A, middle and bottom row contain RGB composite element maps of neutrophils from donor B stimulated for 1 h and 2 h with phorbol myristate acetate (PMA), respectively. All element intensities within the RGB composite element maps are normalized to diode current, dead time and measuring time. Maximum intensity of Ca, Zn and Fe (normalized) counts within the control culture neutrophils were set as upper threshold values for the other RGB composite element maps. Neutrophil nomenclature used in the quantitative analysis is provided within each RGB composite element map. (TIFF) [file pone.0165604.s001.tiff]

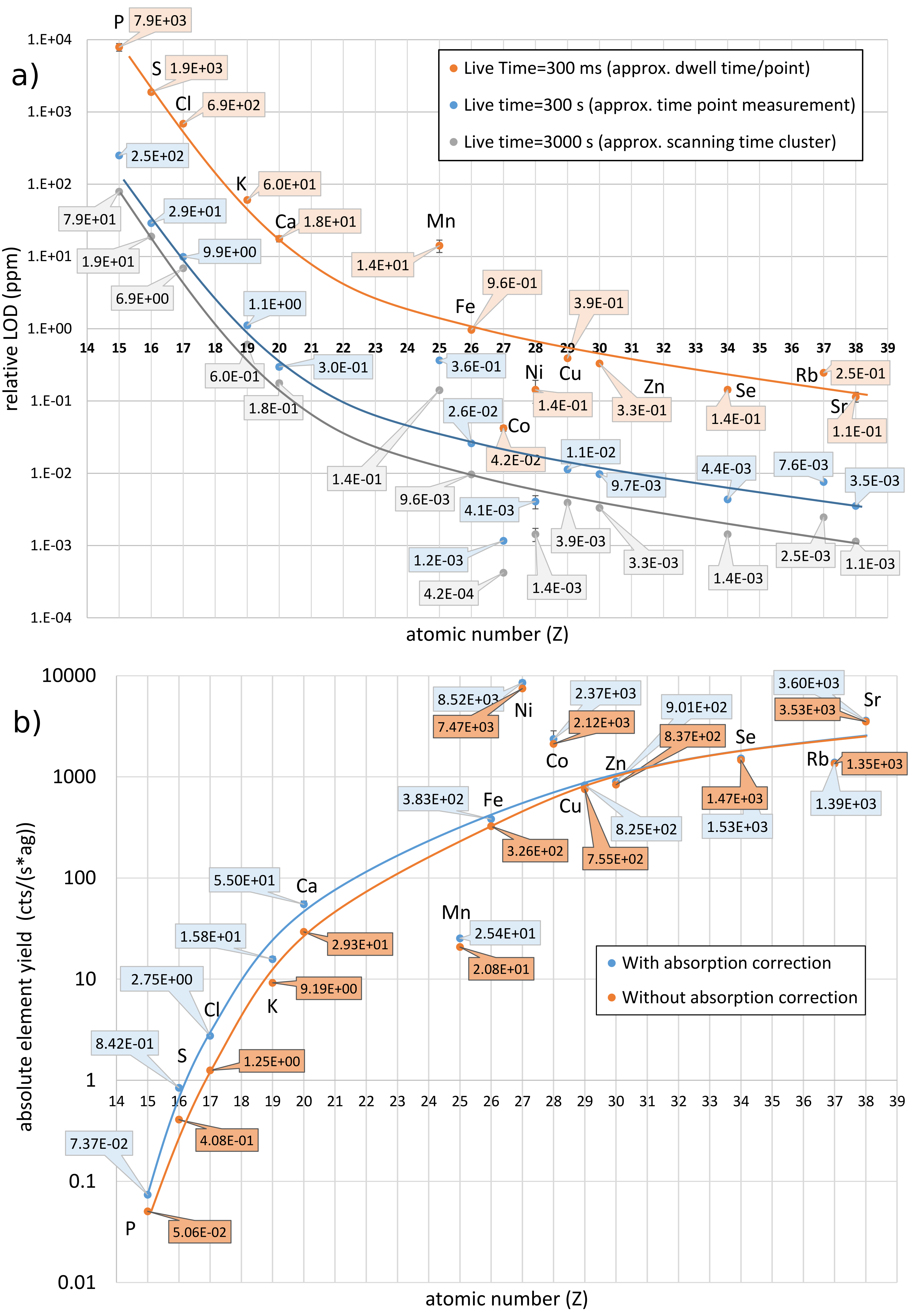

Supplement: S4 Fig — MDLs are shown for a typical dwell time of 300 ms per point in scanning mode, a point measurement of approx. 300 s and a neutrophil sub-area with an approximate scanning time of 3000 s, without beam absorber. Element yields are shown with (blue color) and without (orange color) absorption correction factor. Relative LODs and element yields are both calculated from Kα,total net line and background intensities. Error bars are calculated from relative error of background and net intensity and relative concentration error of NIST SRM 1577C ‘bovine liver’; for more information on this matter, we refer to S1 Text. (TIFF) [file pone.0165604.s004.tiff]

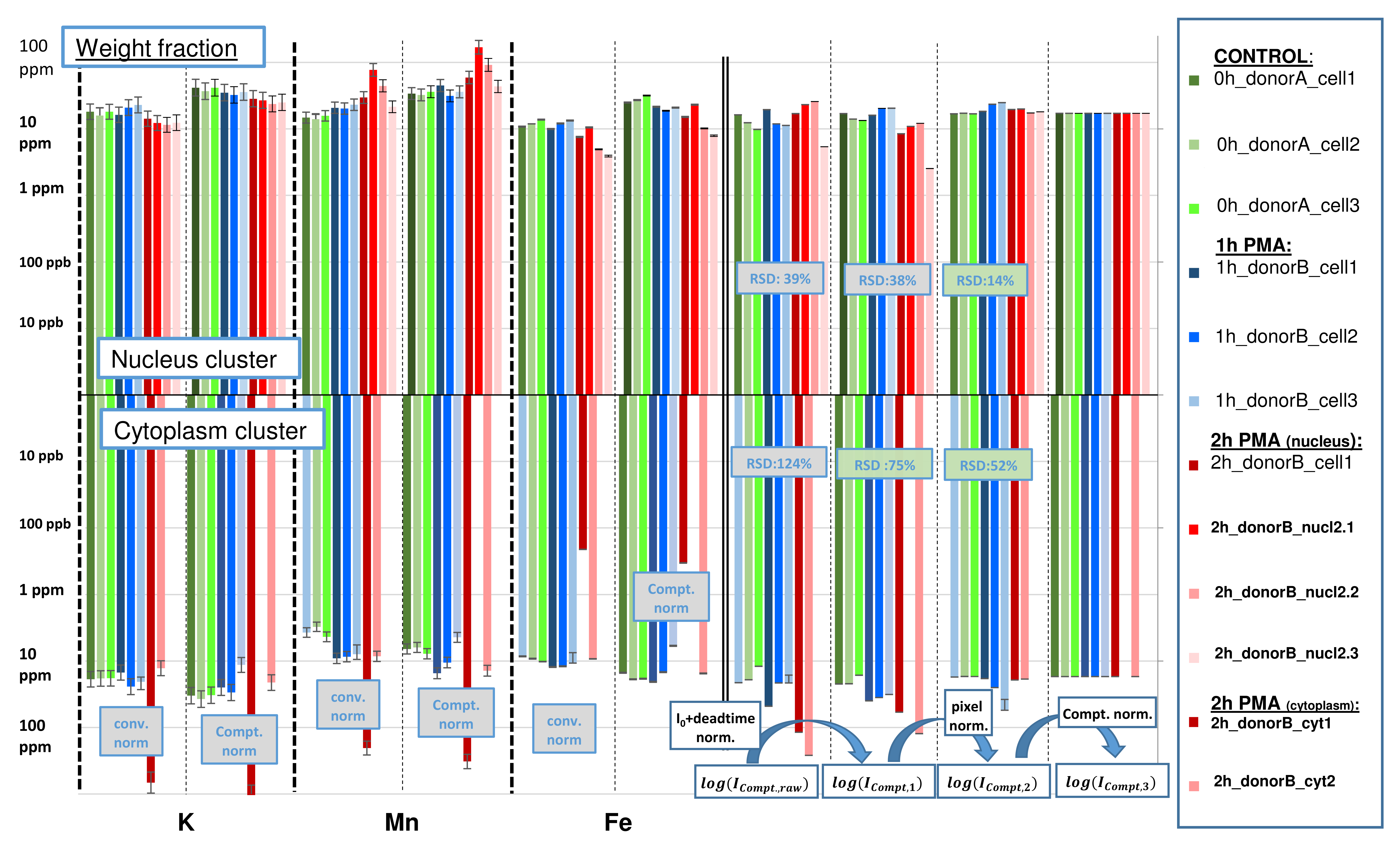

Supplement: S5 Fig — Mass fractions of K, Mn and Fe within neutrophil nuclei and cytoplasms throughout PMA exposure obtained by ‘conventional’ and ‘Compton’ normalization. Right side of double vertical stripe: effect of different normalization steps upon Compton intensity of XRF cluster sum spectra of neutrophil nuclei (above Y-axis) and cytoplasms (below Y-axis). First column: raw cluster sum spectra, 2nd column: raw cluster sum spectra corrected for dead time (DT) and normalized to incoming beam intensity (I0), 3rd column: additional normalization to cluster area (= number of pixels in reference cluster), 4th column: cluster sum spectra normalized to the Compton intensity of the reference cluster. RSD values of nucleus and cytoplasm Compton intensities after each normalization step are shown in square boxes. Left side of double vertical stripe: comparison between K, Mn and Fe mass fractions of neutrophil nuclei (above Y-axis) and cytoplasms (below Y-axis) throughout PMA stimulation, calculated from ‘conventionally’ normalized (left bar) and ‘Compton’ normalized (right bar) element XRF intensities. Quantitative data is expressed in ppm and in logarithmic scale. (TIF) [file pone.0165604.s005.tif]
