# Supplementary material for: Probing Intracellular Element Concentration Changes during Neutrophil Extracellular Trap Formation Using Synchrotron Radiation Based X-Ray Fluorescence
Source: PLoS One. 2016 Nov 3;11(11):e0165604. doi: 10.1371/journal.pone.0165604 (PMC5094720; doi:10.1371/journal.pone.0165604)
Supplement: S1 Text — (DOCX) [file pone.0165604.s010.docx]

### Suppl. Text I: Calculation of the relative error of (absolute) element yield, (relative) limit of detection (LOD), absolute mass and concentration of the analyte. Conversion of the error to log scale.

Relative errors estimations were calculated for: 1) Compton scatter intensities 2) the absolute element yield, 3) the relative limit of detection, 4) total absolute mass of a cluster area (and from here also for mass fraction, molarity) and finally 5) the relative error of the log value of previous quantities. Absolute error bars for the mass fractions are shown in Fig. 3a-b and Fig. 4a-b. Additional error bars for the Compton intensities are shown in Suppl. Fig. 5. Error bars of the element yields/relative LODs are shown in Suppl. Fig. 3.

1. The relative error on the Compton scatter intensity ‘$\delta_{Compt.}$’ is subject to Poisson counting statistics, thus the relative error of N counts equals $\sqrt{N}/N$ (or $1/\sqrt{N}$), therefore is:

$\delta_{Compt.}= \frac{1}{\sqrt{I_{Compt.}}}$ EQ. S1.1

1. The relative error on the element yield ‘$\delta_{yield}$’ of a certain element is calculated from the relative error on the net $K_{\alpha}$ fluorescent intensity ‘$\delta_{N}$’ and the certified relative concentration error ‘$\delta_{conc.}$’ of the NIST SRM mentioned in the specification sheet:

$\delta_{yield}=\sqrt{{\delta_{N}}^{2}+{\delta_{conc.}}^{2}}$ EQ. S1.2

The first term in EQ. S1.1 is governed by Poisson counting statistics as well:

$\delta_{yield}=\sqrt{\left( \frac{1}{\sqrt{I_{K_{\alpha}}}} \right)^{2}{+ \delta_{conc.}}^{2}}$ EQ. S1.3

1. The relative error on the relative LOD ‘$\delta_{LOD}$’ is calculated from the relative error on the background, the relative error on the net intensity and the certified relative error of the concentration:

$\delta_{{LOD}_{relative}}=\sqrt{\left( \frac{1}{2*\sqrt{I_{backgr.}}} \right)^{2}{+\left( \frac{1}{\sqrt{I_{K_{\alpha}}}} \right)}^{2}{+ \delta_{conc.}}^{2}}$ EQ. S1.4

1. The relative error upon the absolute element mass ‘$\delta_{m_{abs.}}$’ within a cluster area ‘${cluster}_{X}$’ is calculated by standard error propagation rules applied onto the relative error of the (absolute) element yield ‘$\delta_{yield}$’ and the relative error of the (normalized) cluster net intensity ‘${\delta(I}_{\mathrm{cluster}_{x}})$’:

$\delta_{m_{abs.}}\left( {cluster}_{X} \right)= \sqrt{{{\delta(I}_{{cluster}_{x}})}^{2}+ {\delta_{yield}}^{2}}$ EQ. S1.5

where ${{\delta(I}_{{cluster}_{x}})}^{2}$ also follows Poisson counting statistics (see Eq.S1.1) and therefore:

$\delta_{m_{abs.}}\left( {cluster}_{X} \right)= \sqrt{\left( \frac{1}{\sqrt{I_{K_{\alpha}}}} \right)^{2}{{+ \delta}_{yield}}^{2}}$ EQ. S1.6

Further on, ${\delta_{yield}}^{2}$is given by Eq. S1.3. As mass fraction (and molarity) are directly derived from the absolute mass, δ values of both these quantities equal ‘$\delta_{m_{abs.}}\left( {cluster}_{X} \right)$’.

1. As the mass fractions of all 15 elements detected covers a vast concentration range (from *%* to *ppb*), a logarithmic scale was used to visualize the mass fractions simultaneously in a bar plot (e.g. as in Fig. 3). Therefore, the relative error δ needs to be recalculated for a logarithmic scale:

$\delta_{log(conc.)}= 0.434 . \delta_{conc.}$ EQ. S1.7.1

The absolute error value $\Delta x$ for log scale depiction is then calculated according to:

${\Delta x}_{\log\left( conc. \right)}=0.434 . \delta_{log(conc.)} .LOG(conc.)$ EQ. S1.7.2
