# Supplementary material for: Probing Intracellular Element Concentration Changes during Neutrophil Extracellular Trap Formation Using Synchrotron Radiation Based X-Ray Fluorescence
Source: PLoS One. 2016 Nov 3;11(11):e0165604. doi: 10.1371/journal.pone.0165604 (PMC5094720; doi:10.1371/journal.pone.0165604)
Supplement: S2 Text — (DOCX) [file pone.0165604.s011.docx]

### Suppl. Text 2: Comparison between mass fraction trend behavior of K, Mn and Fe obtained by conventional and Compton normalization of mass fractions in PMA-stimulated neutrophils.

In this Supplementary Text, we show the effect of all normalization steps upon XRF quantitative values by making use of S5 Fig. *On the right side of the vertical double stripe* (i.e. last four main columns of the graph), we first show the effect of the different normalization steps upon the Compton intensities of the neutrophil nuclei (above Y-axis) and cytoplasms (below Y-axis) throughout PMA exposure. First column shows Compton intensities of the raw cluster spectra, second column Compton intensities corrected for dead time (DT) and normalized to the diode current (I_0_) of a reference spectrum (i.e. from a control culture neutrophil ‘0h_donorA_cell1’), third column are Compton intensities additionally normalized to the reference cluster area (containing 22780 pixels). The RSD values of the Compton intensities of the neutrophils from control culture and 1-2 *h* PMA-exposed culture are recalculated after each normalization step and provided in square boxes. For the Compton intensities of the neutrophil nuclei, normalization to diode current and dead time as described in Eq. 7 in M&M ‘Quantification of neutrophil XRF cluster sum spectra’ largely maintains the relative ratio’s between the different Compton intensities, keeping the RSD value at about 40%. When applying normalization to the cluster area thereafter, we find a (much) lower 15% RSD value for Compton intensities of the nuclei XRF spectra (and for entire neutrophils and embedding resin), whereas for the cytoplasm a 50% RSD value remains. In a fourth (last) column, all Compton intensities of the different neutrophil compartments are set to same reference cluster intensity value of 3.91x10^7^ counts). *On the left side of the double stripe in S5 Fig*, a comparison is made showing the difference between the mass fractions of K, Mn and Fe calculated from element intensities normalized to: I_0_, deadtime and cluster area (i.e. so-called ‘conventional normalization’) and the mass fractions of K, Mn and Fe (additionally) normalized to the Compton intensity of the reference cluster XRF spectrum (i.e. so-called ‘Compton normalization’). For K, we observe a decreasing trend throughout PMA‑stimulation using conventional normalization; after Compton normalization, 1 *h* PMA-exposed neutrophils are even better obeying this trend. For Mn, a clear increasing concentration trend can be observed in conventional normalization mode; after Compton normalization the Mn concentration trend is still increasing but with more spread. For Fe, slight changes are present between both normalization methods, but do not have a large influence upon the general concentration trends.
